# Supplementary material for: Discovery of a chemical probe for PRDM9
Source: Nat Commun. 2019 Dec 17;10:5759. doi: 10.1038/s41467-019-13652-x (PMC6917776; doi:10.1038/s41467-019-13652-x)
Supplement: Supplementary file 1 — Supplementary Information [file 41467_2019_13652_MOESM1_ESM.pdf]

Supplementary Information

Discovery of a Chemical Probe for PRDM9

Abdellah Allali-Hassani[1#], Magdalena Szewczyk[1#], Danton Ivanochko[1,2#], Shawna Organ[1], Jabez Bok[3], Jessica Sook Yuin Ho[3], Florence P.H Gay[3], Fengling Li[1], Levi Blazer[1], Mohammad S. Eram[1], Levon Halabelian[1], David Dilworth[1], Genna M. Luciani[1], Evelyne Lima-Fernandes[1], Qin Wu[1], Peter Loppnau[1], Nathan Palmer[3], S. Zakiah A. Talib[3], Peter J. Brown[1], Matthieu Schapira[1,4], Philipp Kaldis[3, 5], Ronan C. O'Hagan[6], Ernesto Guccione[3, 7, 8], Dalia Barsyte-Lovejoy[1,9], Cheryl H. Arrowsmith[1,2], John M. Sanders[6], Solomon D. Kattar[6], D. Jonathan Bennett[6], Benjamin Nicholson[6]* and Masoud Vedadi[1,4]*

[1]Structural Genomics Consortium, University of Toronto, Toronto, ON, M5G 1L7, Canada

[2]Princess Margaret Cancer Centre and Department of Medical Biophysics, University of Toronto, Toronto, ON, M5G 2M9, Canada.

[3]Institute of Molecular and Cell Biology (IMCB), Agency for Science, Technology and Research (A*STAR), Singapore.

[4]Department of Pharmacology and Toxicology, University of Toronto, Toronto, ON, M5S 1A8, Canada

[5]National University of Singapore (NUS), Department of Biochemistry,117597, Singapore.

[6]Merck & Co., Inc., 2000 Galloping Hill Road, Kenilworth, New Jersey 07033, United States.

[7]Department of Oncological Sciences and Tisch Cancer Institute, Icahn School of Medicine at Mount Sinai, New York, NY 10029, USA.

[8]Department of Pharmacological Sciences and Mount Sinai Center for Therapeutics Discovery, Icahn School of Medicine at Mount Sinai, New York, NY 10029, USA.

[9]Nature Research Center, Vilnius, Akademijos 2, Lithuania.

*Corresponding Authors:
Masoud Vedadi; m.vedadi@utoronto.ca; Tel: 416 432 1980
Benjamin Nicholson; benjamin.nicholson@merck.com; Tel: 617 992 3213

[#] equally contributed to this manuscript.

Table of content

Supplementary Table 1. Crystallographic data collection and refinement statistics. Page 3

Supplementary Table 2. Atoms tallied at the interface between SAM or SAH and small molecule inhibitors. Page 4

Supplementary Table 3. Primer sequences and coordinates for ChIP-PCR assay. Page 5

Supplementary Figure 1. Protein methyltransferase phylogenetic trees. Page 6

Supplementary Figure 2. Discovery of MRK-740. Page 7

Supplementary Figure 3. Assessing the binding of MRK-740 to PRDM9 by differential scanning fluorimetry (DSF). Page 7

Supplementary Figure 4. Methyltransferase selectivity of MRK-740 and MRK-740-NC. Page 8

Supplementary Figure 5. Assessment of GPCR functional activity. Page 9

Supplementary Figure 6. Effect of SAH on MRK-740 binding. Page 10

Supplementary Figure 7. MRK-740 fitting of the electron density map. Page 12

Supplementary Figure 8. WaterMap hydration sites for SAM versus SAH in the presence of MRK-740. Page 13

Supplementary Figure 9. MRK-740 inhibits PRDM9-dependent lysine trimethylation of endogenous histone H3. Page 14

Supplementary Figure 10. The effect of MRK-740 on cell growth Page 15

Supplementary Figure 11. MRK-740 inhibits PRDM9 mediated methylation of exogenous histone H3 in MCF7 cells Page 16

Supplementary Figure 12. MRK-740 selectively depletes PRDM9-dependent H3K4me3 formation. Page 17

Supplementary Figure 13. Effect of MRK-740 on viability of cancer cell lines. Page 19

Supplementary Figure 14. Comparison of meiotic progression in the presence of MRK-NC and MRK470 treated spermatocytes. Page 21

Supplementary Methods Page 22

Supplementary Table 1. Crystallographic data collection and refinement statistics.

	6NM4
**Data collection**	
Space group	P 21 21 21
Wavelength (Å)	0.97918
Cell dimensions	
a, b, c (Å)	38.03, 74.80, 141.44
α, β, γ (°)	90, 90, 90
Resolution (Å)	39.88 – 2.58 (2.69 – 2.58)
$R_{merge\ (\%)}$	0.13 (1.65)
$I / \sigma I$	10.7 (1.3)
Completeness (%)	99.6 (99.4)
Redundancy	7.3 (6.3)
**Refinement**	
Resolution (Å)	39.88 – 2.58
No. reflections	13297
R_{work} / R_{free}	0.2088/0.2588
Wilson B factor (Å^2)	61.6
No. atoms	
Protein	2924
Ligand/ion	124
Water	23
Unidentified	3
B-factor (Å^2)	
Average	63.1
Macromolecules	63.0
Ligands	65.8
R.m.s. deviations	
Bond lengths (Å)	0.0104
Bond angles (°)	1.471

Values in parentheses are for highest-resolution shell.

Supplementary Table 2. Atoms tallied at the interface between SAM or SAH and small molecule inhibitors. The data are presented in Figure 4. This table has also been provided as "Supplementary data 4" in excel.

pdb_id	LIGAND_name	SAMSAH_atoms	LIGAND_atoms	LIGAND_ID	family_ID	Uniprot.Recommended.Name	Gene.Name	Taxonomy
1h1d	BIA	5	7	BIA	COMT	Catechol O-methyltransferase	Comt	Rattus norvegicus
1hnn	SKF	3	2	SKF	PNMT	Phenylethanolamine N-methyltransferase	PNMT	Homo sapiens
1jqe	QUN	18	10	QUN	Rossmann-type	Histamine N-methyltransferase	HNMT	Homo sapiens
1n7i	LY1	3	3	LY1	PNMT	Phenylethanolamine N-methyltransferase	PNMT	Homo sapiens
1n7j	IDI	5	2	IDI	PNMT	Phenylethanolamine N-methyltransferase	PNMT	Homo sapiens
1vid	DNC	5	9	DNC	COMT	Catechol O-methyltransferase	Comt	Rattus norvegicus
2an3	CTL	2	2	CTL	PNMT	Phenylethanolamine N-methyltransferase	PNMT	Homo sapiens
2an5	TTL	2	1	TTL	PNMT	Phenylethanolamine N-methyltransferase	PNMT	Homo sapiens
2cl5	BIE	10	9	BIE	COMT	Catechol O-methyltransferase	Comt	Rattus norvegicus
2g70	HNT	7	10	HNT	PNMT	Phenylethanolamine N-methyltransferase	PNMT	Homo sapiens
2g71	FTS	3	3	FTS	PNMT	Phenylethanolamine N-methyltransferase	PNMT	Homo sapiens
2g72	F21	7	10	F21	PNMT	Phenylethanolamine N-methyltransferase	PNMT	Homo sapiens
2g8n	F83	3	3	F83	PNMT	Phenylethanolamine N-methyltransferase	PNMT	Homo sapiens
2obf	F83	3	3	F83	PNMT	Phenylethanolamine N-methyltransferase	PNMT	Homo sapiens
2ony	TMJ	2	2	TMJ	PNMT	Phenylethanolamine N-methyltransferase	PNMT	Homo sapiens
2onz	TMJ	3	2	TMJ	PNMT	Phenylethanolamine N-methyltransferase	PNMT	Homo sapiens
2opb	F21	4	4	F21	PNMT	Phenylethanolamine N-methyltransferase	PNMT	Homo sapiens
2y1x	845	7	7	845	Rossmann-type	Histone-arginine methyltransferase CARM1	PRMT4	Homo sapiens
2zvj	KOM	7	8	KOM	COMT	Catechol O-methyltransferase	Comt	Rattus norvegicus
3a7e	DNC	8	8	DNC	COMT	Catechol O-methyltransferase	COMT	Homo sapiens
3bwm	DNC	7	8	DNC	COMT	Catechol O-methyltransferase	COMT	Homo sapiens
3bwy	DNC	6	8	DNC	COMT	Catechol O-methyltransferase	COMT	Homo sapiens
3k5k	DXQ	1	2	DXQ	SET-type	Histone-lysine N-methyltransferase EHMT2	EHMT2	Homo sapiens
3kpu	ES1	3	2	ES1	PNMT	Phenylethanolamine N-methyltransferase	PNMT	Homo sapiens
3kpy	ES2	5	2	ES2	PNMT	Phenylethanolamine N-methyltransferase	PNMT	Homo sapiens
3kqo	ES4	6	3	ES4	PNMT	Phenylethanolamine N-methyltransferase	PNMT	Homo sapiens
3kqp	ES5	3	2	ES5	PNMT	Phenylethanolamine N-methyltransferase	PNMT	Homo sapiens
3kqq	ES6	2	1	ES6	PNMT	Phenylethanolamine N-methyltransferase	PNMT	Homo sapiens
3kqs	AX7	6	4	AX7	PNMT	Phenylethanolamine N-methyltransferase	PNMT	Homo sapiens
3kqt	ES7	4	2	ES7	PNMT	Phenylethanolamine N-methyltransferase	PNMT	Homo sapiens
3kqv	FAN	2	1	FAN	PNMT	Phenylethanolamine N-methyltransferase	PNMT	Homo sapiens
3kqw	ES9	4	1	ES9	PNMT	Phenylethanolamine N-methyltransferase	PNMT	Homo sapiens
3kqy	ES0	6	3	ES0	PNMT	Phenylethanolamine N-methyltransferase	PNMT	Homo sapiens
3kr1	VGD	5	2	VGD	PNMT	Phenylethanolamine N-methyltransferase	PNMT	Homo sapiens
3kr2	ET2	4	2	ET2	PNMT	Phenylethanolamine N-methyltransferase	PNMT	Homo sapiens
3mo2	E67	3	3	E67	SET-type	Histone-lysine N-methyltransferase EHMT1	EHMT1	Homo sapiens
3mo5	E72	4	3	E72	SET-type	Histone-lysine N-methyltransferase EHMT1	EHMT1	Homo sapiens
3rjw	CIQ	3	2	CIQ	SET-type	Histone-lysine N-methyltransferase EHMT2	EHMT2	Homo sapiens
3s68	TCW	7	10	TCW	COMT	Catechol O-methyltransferase	Comt	Rattus norvegicus
3s7b	NH5	4	6	NH5	SET-type	N-lysine methyltransferase SMYD2	SMYD2	Homo sapiens
4.00E+47	0N6	6	5	0N6	SET-type	Histone-lysine N-methyltransferase SETD7	SETD7	Homo sapiens
4jds	1L4	6	5	1L4	SET-type	Histone-lysine N-methyltransferase SETD7	SETD7	Homo sapiens
4jlg	1L8	7	7	1L8	SET-type	Histone-lysine N-methyltransferase SETD7	SETD7	Homo sapiens
4nvq	2OD	1	2	2OD	SET-type	Histone-lysine N-methyltransferase EHMT2	EHMT2	Homo sapiens
4wuy	3UJ	5	3	3UJ	SET-type	N-lysine methyltransferase SMYD2	SMYD2	Homo sapiens
4x61	3XV	14	14	3XV	Rossmann-type	Protein arginine N-methyltransferase 5	PRMT5	Homo sapiens
4x63	3XV	10	7	3XV	Rossmann-type	Protein arginine N-methyltransferase 5	PRMT5	Homo sapiens
4xuc	43G	7	5	43G	COMT	Catechol O-methyltransferase	COMT	Homo sapiens
4xud	43H	9	7	43H	COMT	Catechol O-methyltransferase	COMT	Homo sapiens
4xue	43J	9	11	43J	COMT	Catechol O-methyltransferase	COMT	Homo sapiens
4y2h	49K	5	4	49K	Rossmann-type	Protein arginine N-methyltransferase 6	PRMT6	Homo sapiens
4y30	49L	4	6	49L	Rossmann-type	Protein arginine N-methyltransferase 6	PRMT6	Homo sapiens
4ynd	4GQ	4	5	4GQ	SET-type	N-lysine methyltransferase SMYD2	SMYD2	Homo sapiens
5arf	I9H	3	2	I9H	SET-type	N-lysine methyltransferase SMYD2	SMYD2	Homo sapiens
5arg	H41	4	2	H41	SET-type	N-lysine methyltransferase SMYD2	SMYD2	Homo sapiens
5ayf	C7H	5	5	C7H	SET-type	Histone-lysine N-methyltransferase SETD7	SETD7	Homo sapiens
5ccl	4ZW	4	4	4ZW	SET-type	Histone-lysine N-methyltransferase SMYD3	SMYD3	Homo sapiens
5ccm	4ZX	4	4	4ZX	SET-type	Histone-lysine N-methyltransferase SMYD3	SMYD3	Homo sapiens
5cpr	539	3	2	539	SET-type	Histone-lysine N-methyltransferase KMT5B	SUV420H1	Homo sapiens
5e8r	5L6	7	3	5L6	Rossmann-type	Protein arginine N-methyltransferase 6	PRMT6	Homo sapiens
5egs	5NR	5	4	5NR	Rossmann-type	Protein arginine N-methyltransferase 6	PRMT6	Homo sapiens
5eml	5QK	12	14	5QK	Rossmann-type	Protein arginine N-methyltransferase 5	PRMT5	Homo sapiens
5fhq	DNC	7	8	DNC	COMT	Catechol O-methyltransferase	Comt	Rattus norvegicus
5fhr	DNC	9	6	DNC	COMT	Catechol O-methyltransferase	Comt	Rattus norvegicus
5kjk	6T1	7	6	6T1	SET-type	N-lysine methyltransferase SMYD2	SMYD2	Homo sapiens
5kjm	6TM	7	7	6TM	SET-type	N-lysine methyltransferase SMYD2	SMYD2	Homo sapiens
5kjn	6TL	7	9	6TL	SET-type	N-lysine methyltransferase SMYD2	SMYD2	Homo sapiens
5lsa	DNC	7	7	DNC	COMT	Catechol O-methyltransferase	COMT	Homo sapiens
5p9o	7JF	7	5	7JF	COMT	Catechol O-methyltransferase	Comt	Rattus norvegicus
5p9p	7JG	7	5	7JG	COMT	Catechol O-methyltransferase	Comt	Rattus norvegicus
5p9q	7JH	7	4	7JH	COMT	Catechol O-methyltransferase	Comt	Rattus norvegicus
5p9r	7JJ	6	4	7JJ	COMT	Catechol O-methyltransferase	Comt	Rattus norvegicus
5p9s	7JK	6	4	7JK	COMT	Catechol O-methyltransferase	Comt	Rattus norvegicus
5p9t	7JD	7	4	7JD	COMT	Catechol O-methyltransferase	Comt	Rattus norvegicus
5p9u	7JL	7	4	7JL	COMT	Catechol O-methyltransferase	Comt	Rattus norvegicus
5p9v	7JM	7	4	7JM	COMT	Catechol O-methyltransferase	Comt	Rattus norvegicus
5p9z	7JQ	7	5	7JQ	COMT	Catechol O-methyltransferase	Comt	Rattus norvegicus
5pa0	7JR	5	5	7JR	COMT	Catechol O-methyltransferase	Comt	Rattus norvegicus
5pa1	7JS	7	5	7JS	COMT	Catechol O-methyltransferase	Comt	Rattus norvegicus
5pa2	7JU	7	4	7JU	COMT	Catechol O-methyltransferase	Comt	Rattus norvegicus
5pa3	7JV	7	4	7JV	COMT	Catechol O-methyltransferase	Comt	Rattus norvegicus
5pa4	7JD	7	4	7JD	COMT	Catechol O-methyltransferase	Comt	Rattus norvegicus
5pa5	7JH	7	4	7JH	COMT	Catechol O-methyltransferase	Comt	Rattus norvegicus
5pa6	7JW	7	5	7JW	COMT	Catechol O-methyltransferase	Comt	Rattus norvegicus
5pa7	7JX	7	4	7JX	COMT	Catechol O-methyltransferase	Comt	Rattus norvegicus
5u4x	7VM	7	4	7VM	Rossmann-type	Histone-arginine methyltransferase CARM1	PRMT4	Homo sapiens
5v37	8WD	6	7	8WD	SET-type	Histone-lysine N-methyltransferase SMYD3	SMYD3	Homo sapiens
5v3h	8WG	4	6	8WG	SET-type	N-lysine methyltransferase SMYD2	SMYD2	Homo sapiens
5wbv	9ZY	4	5	9ZY	SET-type	Histone-lysine N-methyltransferase KMT5B	SUV420H1	Homo sapiens
5xxd	8NR	6	7	8NR	SET-type	Histone-lysine N-methyltransferase SMYD3	SMYD3	Homo sapiens
5xxg	8HR	4	7	8HR	SET-type	Histone-lysine N-methyltransferase SMYD3	SMYD3	Homo sapiens
5xxj	8HF	4	7	8HF	SET-type	Histone-lysine N-methyltransferase SMYD3	SMYD3	Homo sapiens
5yjo	8W0	4	5	8W0	SET-type	Histone-lysine N-methyltransferase SMYD3	SMYD3	Homo sapiens
6arj	BW4	7	16	BW4	Rossmann-type	Histone-arginine methyltransferase CARM1	PRMT4	Homo sapiens
6arv	BW7	7	6	BW7	Rossmann-type	Histone-arginine methyltransferase CARM1	PRMT4	Homo sapiens
6gy1	FGQ	6	5	FGQ	COMT	Catechol O-methyltransferase	Comt	Rattus norvegicus
6nm4	KS7	27	17	KS7	SET-type	Histone-lysine N-methyltransferase PRDM9	PRDM9	Homo sapiens

Supplementary Table 3. Primer sequences and coordinates for ChIP-PCR assay

Chr	Start	End	Gene	Primer F	Primer R
chr3	165515070	165515087	BCHE	AGGAGATACGAGGCAATGTTTAG	GAAAGAAGGAAGGAAGGGAAAGA
chr2	40281734	40281748	SLC8A1-AS1	CCCAGCTATGTTCACAGAAGAA	CCTGGAGCAAGATGAACAGAA
chr2	128812120	128812141	intergenic	ACTTCTCCTGCAAACAGACC	CCAATCCCAACCACAGAAGA
chr8	49932798	49932810	intergenic	CCTACTCCAACAGAGACACATC	GAGCTTCAGGGTGGAGATAAA
chr19	8510167	8510261	HNRNPM	GGGAGCCTCCAGAATCGT	GCCTGAGGCCTCGACTA
chr12	6643945	6644023	GAPDH	TAGGCGCTCACTGTTCTCT	TGACTCCGACCTTCACCTT

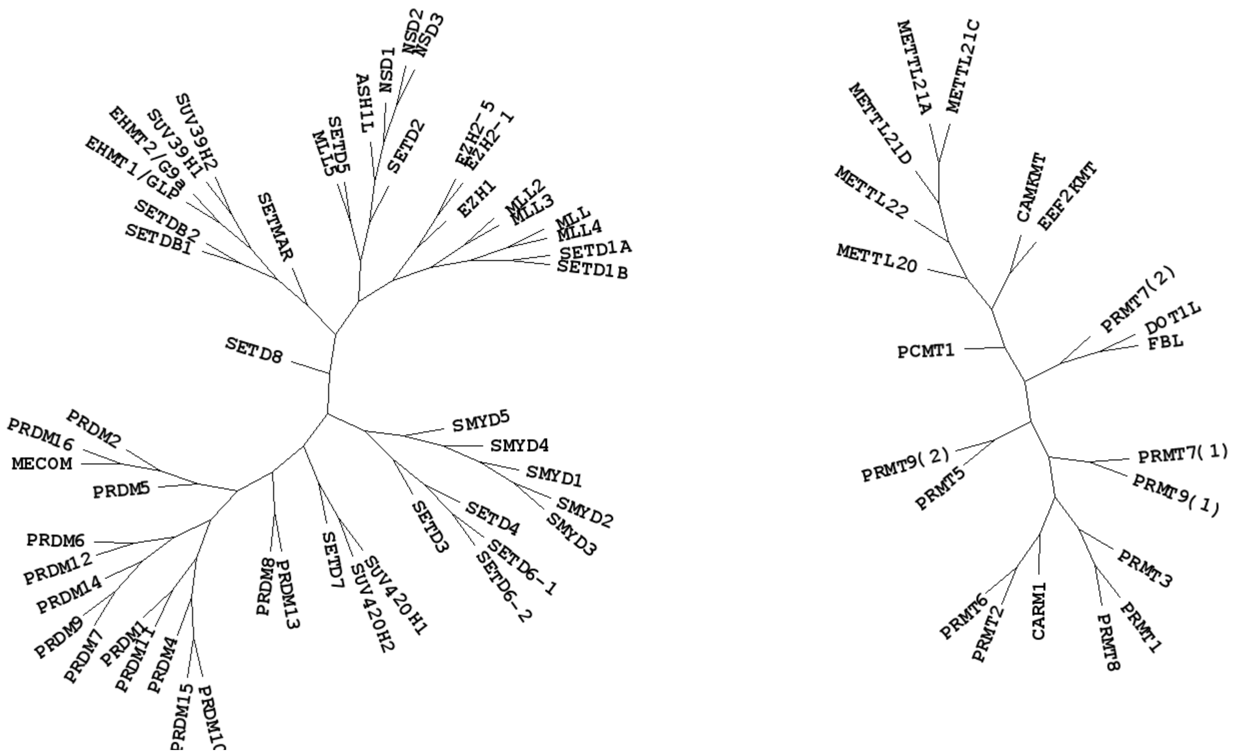

Supplementary Figure 1. Protein methyltransferases phylogenetic trees. Phylogenetic trees for protein lysine methyltransferases (left) and arginine methyltransferases (right) are presented. The figures are from SGC website:
http://apps.thesgc.org/resources/phylogenetic_trees//?domain=HMT#options

The phylogeny outlined in the tree is derived from a multiple sequence alignment of the PMT domain. If a domain is present multiple times in a protein, the protein is shown multiple times in the tree, followed by the sequential iteration of the domain in parenthesis: for example, PRMT7(2) corresponds to the second PMT domain of the protein PRMT7. If multiple variants with insertions or deletions were reported for a gene, the variant number according to Swiss-Prot nomenclature is indicated after a hyphen: for example, EZH2-2 corresponds to the second Swiss-Prot variant of the EZH2 gene. A seed alignment was derived from available protein structures by aligning residues that were superimposed in the three-dimensional space. Additional sequences were appended by aligning them to the closest seed sequence.

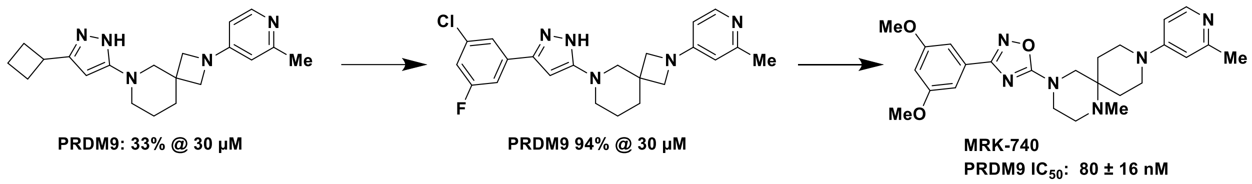

Supplementary Figure 2. Discovery of MRK-740. Through screening a library of 7500 compounds, we identified a suitable hit for further studies (the first compound on the left). Initial limited SAR campaign resulted in identifying a more potent compound (the compound in the middle). Extensive follow up SAR resulted in discovery of MRK-740 as a potent PRDM9 inhibitor (the compound on the right). Structures are provided in ChemDraw as Supplementary Data 2.

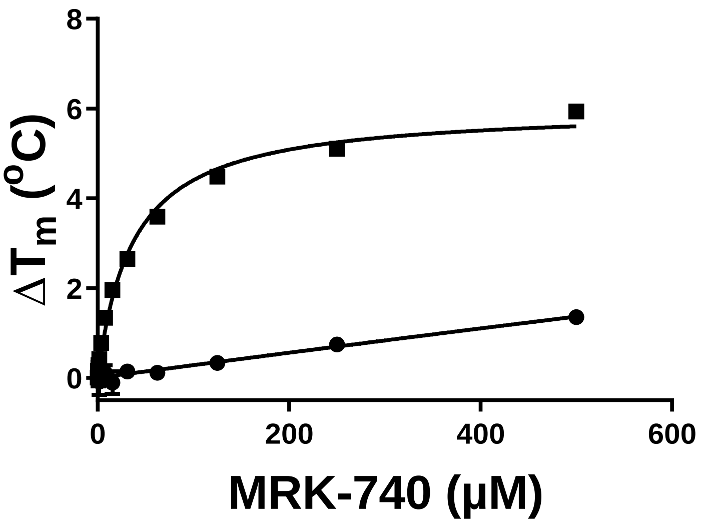

Supplementary Figure 3. Assessing the binding of MRK-740 to PRDM9 by differential scanning fluorimetry (DSF). Titration of PRDM9 by MRK-740 in the (square) presence and (circle) absence of 2 mM SAM. Experiments were performed in triplicate (n=3) and plotted values are the average of 3 replicate ± standard deviation. Source data are provided as a Source Data file.

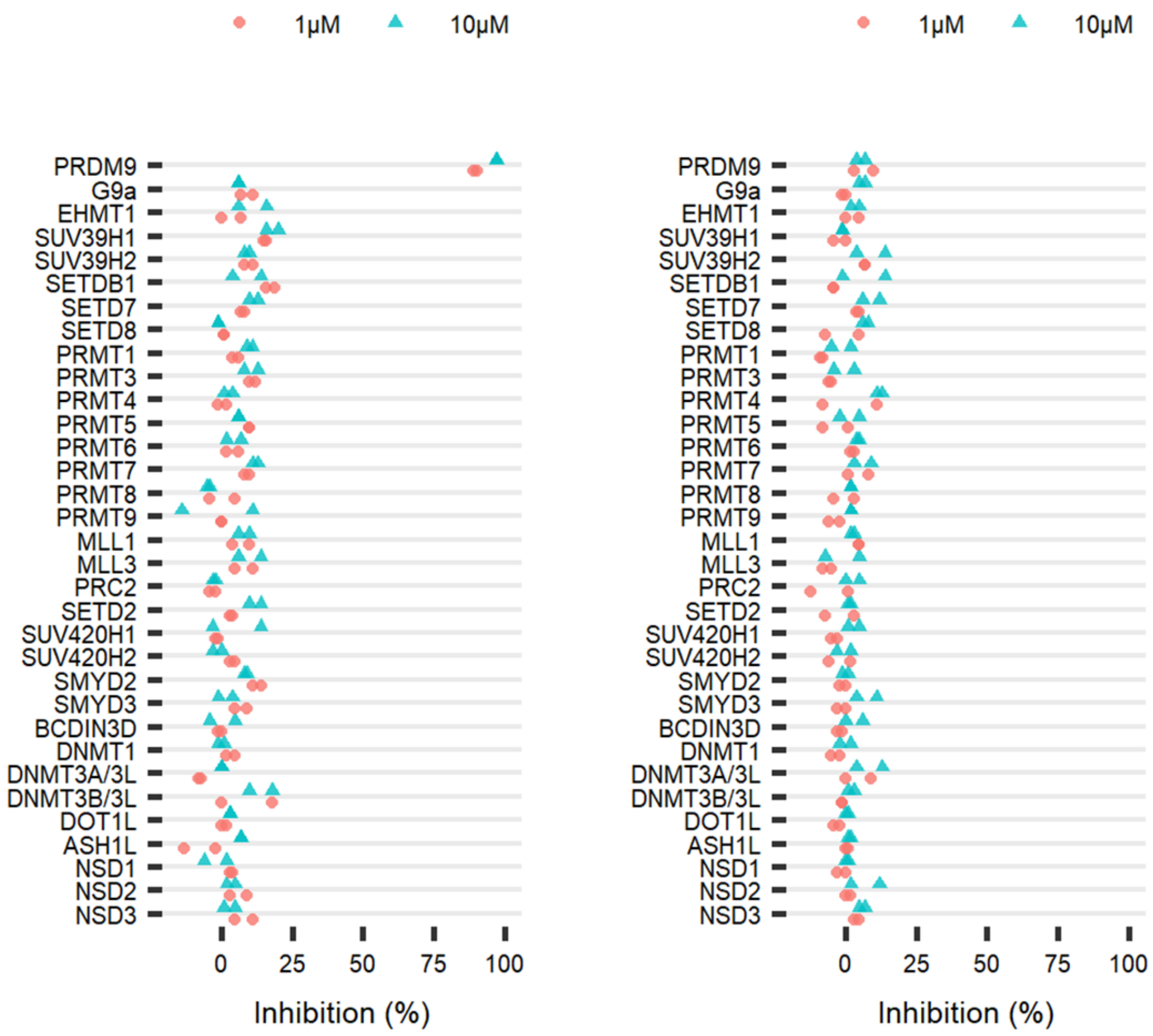

Supplementary Figure 4. Methyltransferase selectivity of MRK-740 and MRK-740-NC. Selectivity of MRK-740 (left) and MRK-740-NC (right) against 32 methyltransferases (MTs) were tested at (circle) 1 µM and (triangle) 10 µM of compounds. PRDM9 was the only methyltransferase in this panel of MTs that was inhibited by MRK-740. MRK-740-NC showed no effect on activity of any of 33 MTs. Effect of both compounds on PRDM7 is presented in figure 1D in the main text. Each experiment was performed at two concentrations (1 and 10 µM) of compounds and in duplicate (n=2). Source data are provided as a Source Data file.

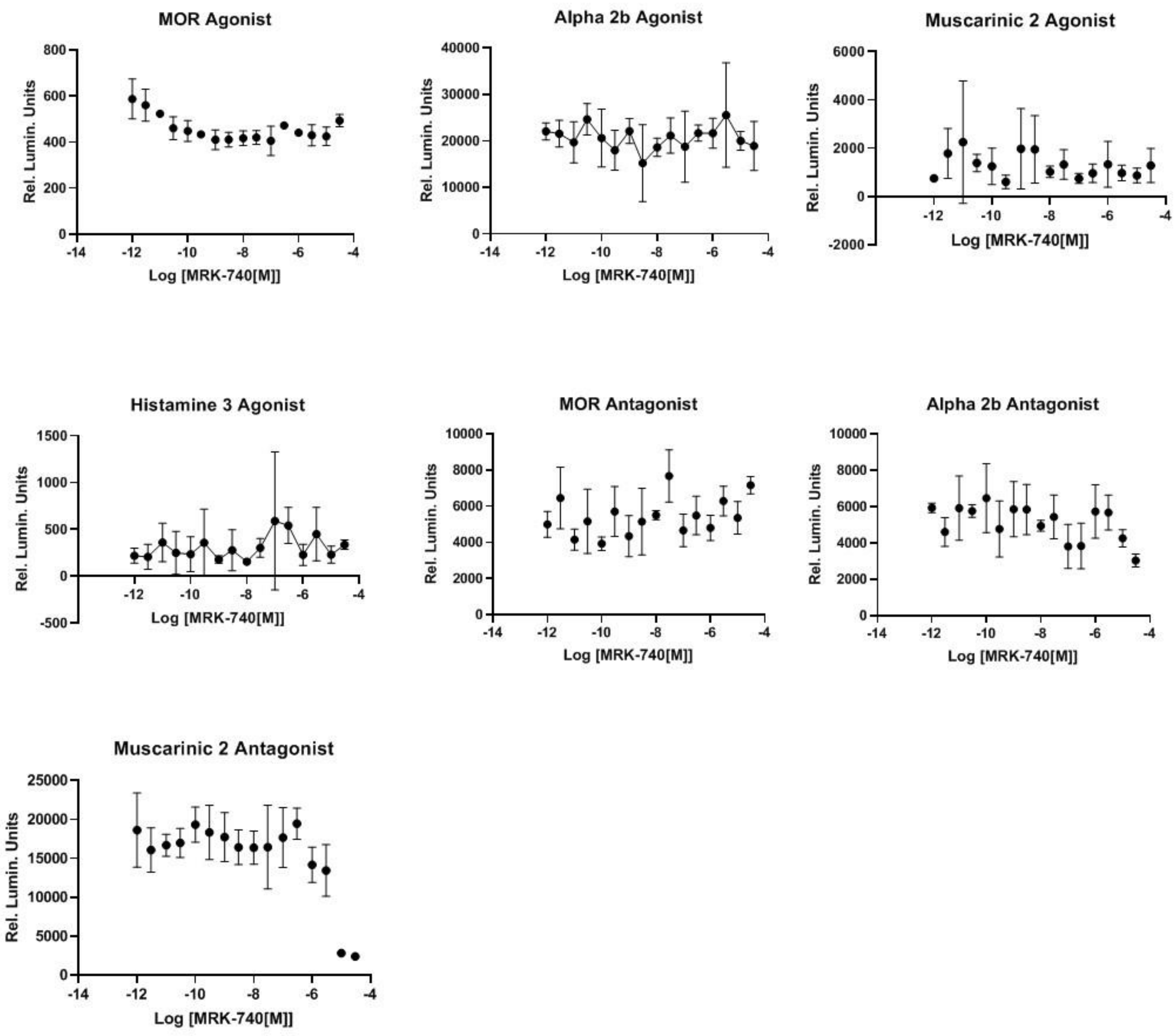

Supplementary Figure 5. Assessment of GPCR functional activity. GloSensor® assay was used for the four targets which showed significant GPCR radioligand binding. GPCR functional profiles were generously provided by the National Institute of Mental Health's (NIMH) Psychoactive Drug Screening Program, (Contract # HHSN-271-2013-00017-C) directed by B.L. Roth, University of North Carolina at Chapel Hill and Project Officer J. Driscoll at NIMH, Bethesda, Maryland, USA. Further details available at https://pdspdb.unc.edu/pdspWeb/. MOR stands for "Opiate μ receptor". Experiments were performed in triplicate (n=3), and plotted values are the average of 3 replicate ± standard deviation. Source data are provided as a Source Data file.

a

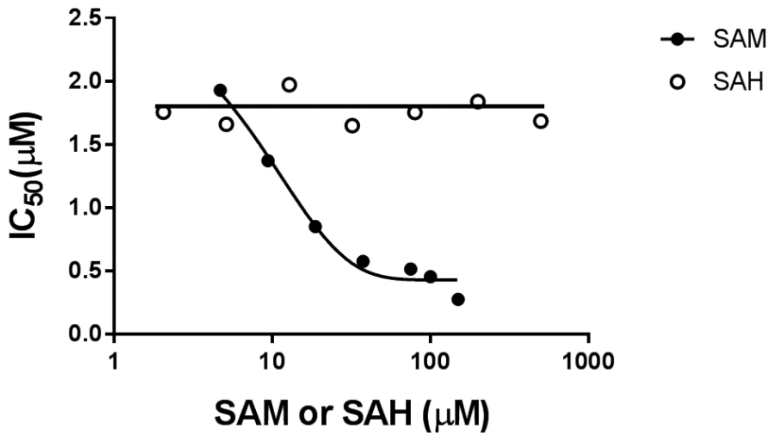

b

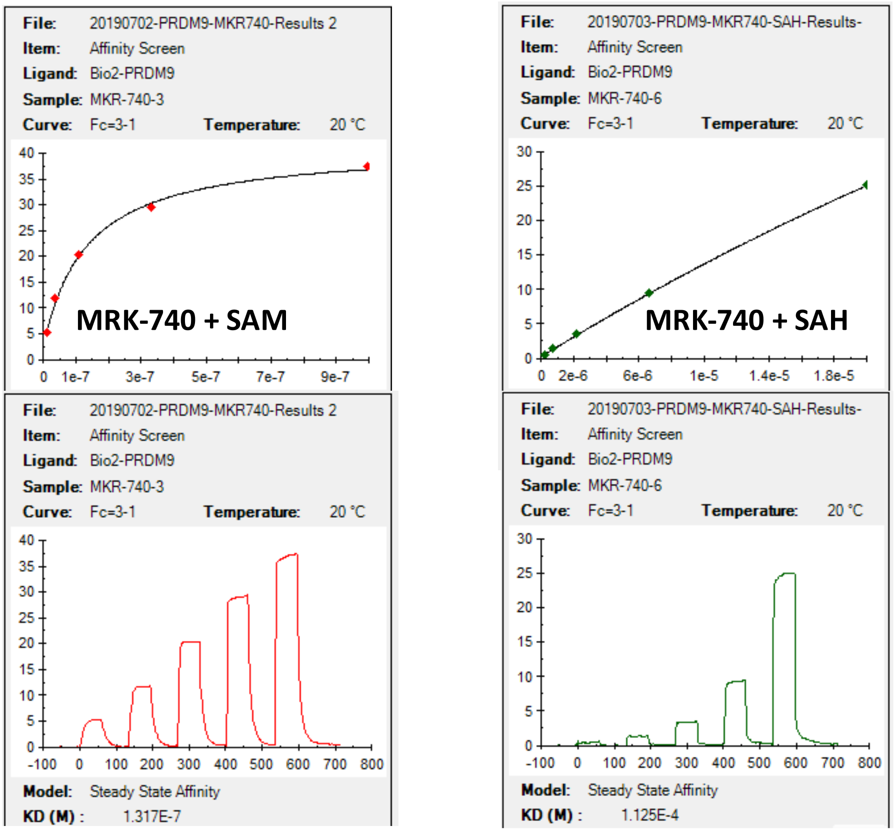

Supplementary Figure 6. Effect of SAH on MRK-740 binding. (a) Experiments in figure 2d were performed at increasing concentrations of SAH in parallel to SAM. No synergistic inhibitory effect was observed for SAH. (b) We also performed SPR for binding of MRK-740 in

the presence of SAH in parallel with SAM indicating that the binding of MRK-740 to PRDM9 was dramatically weaker in the presence of SAH. The data for binding of MRK-740 to PRDM9 in the presence of SAM are the same as presented in figure 2 (a and b) within the main text. The experiments were performed in quadruplicate in the presence of 350 µM SAM (5x K_m) or 750 µM SAH (5x IC_{50}). Biotinylated PRDM9 (195-415aa) was immobilized on the flow cell of an SA sensor chip in 1x HBS-EP buffer, yielding 5700 RU. Using the buffer with 0.5% DMSO, 350 µM SAM or 750 µM SAH and single cycle kinetics with 60 s contact time and a dissociation time of 120 s at a flow rate of 75 µL/min. MRK-740 was tested at 1 µM in the presence of SAM and at 20 µM in the presence of SAH as the highest concentration. Dilution factor of 0.33 was used to yield 5 concentrations.

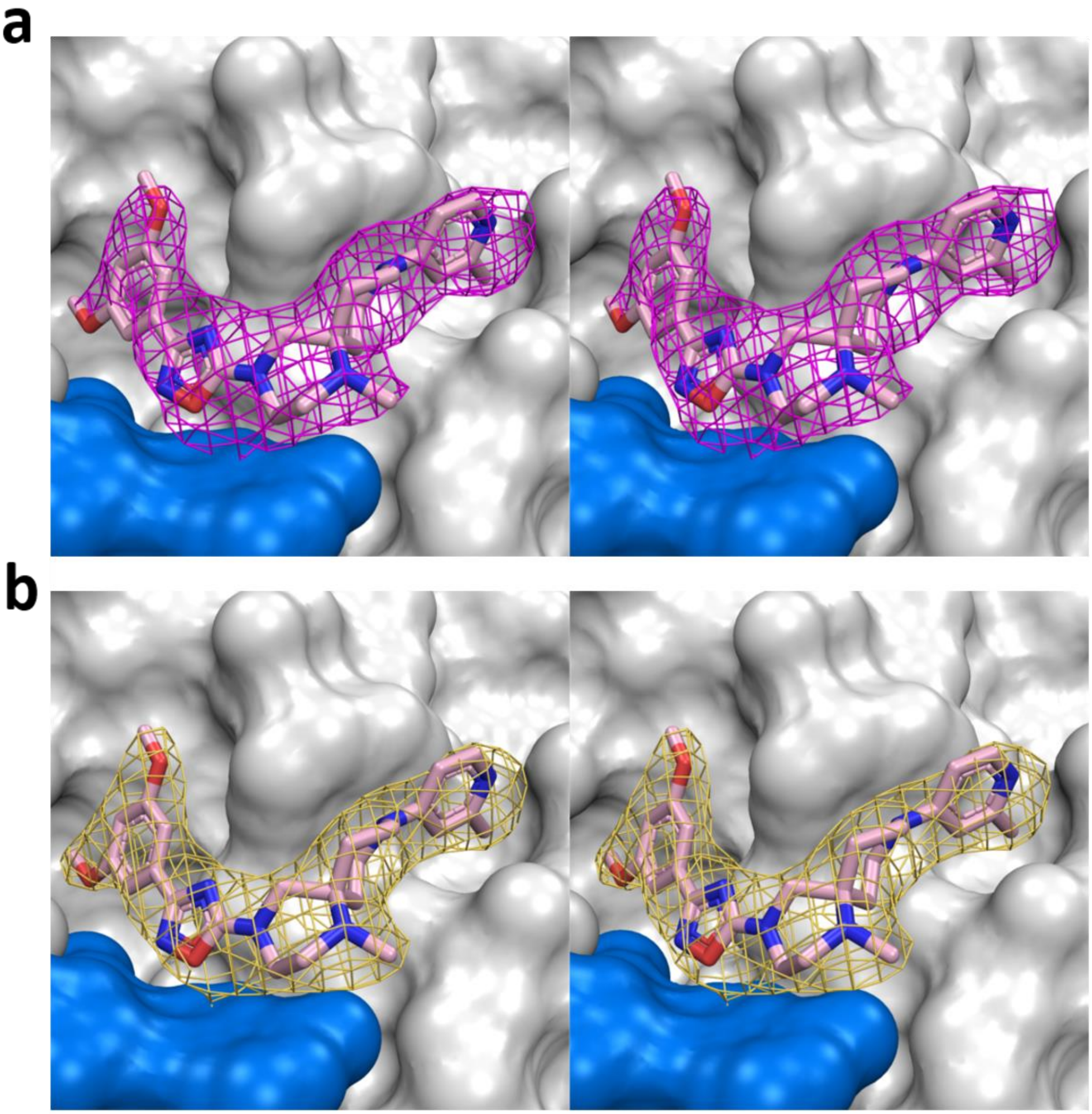

Supplementary Figure 7. Stereo images of MRK-740 fitting to the electron density. Surface representation of PRDM9 PR-SET domain (white) bound by SAM (blue) and MRK-740 showing the (a) Fo – Fc difference electron density map and (b) the refined 2Fo – Fc electron density map of MRK-740 contoured at 2.5 σ (purple) and 1.0 σ (yellow), respectively.

a

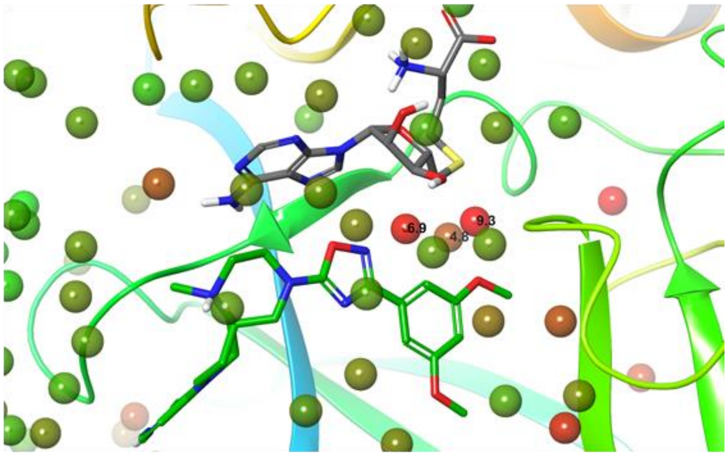

b

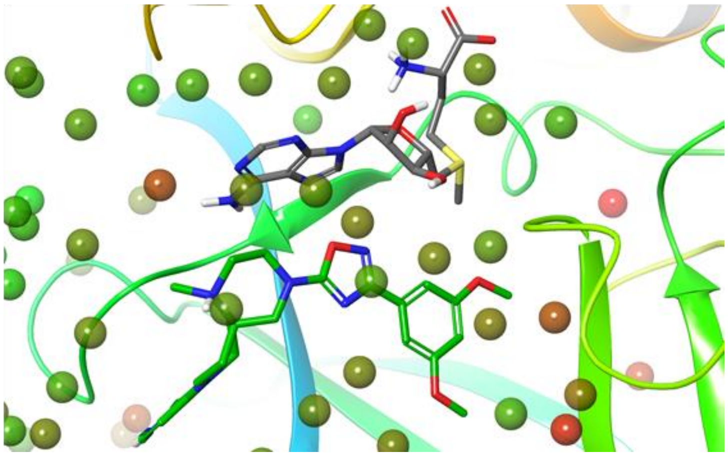

Supplementary Figure 8. WaterMap hydration sites for SAM versus SAH in the presence of MRK-740. (a) WaterMap hydration sites for the PRDM9/SAH/MRK-740 complex. Three hydration sites were identified in the pocket between PRDM9, SAH, and MRK-740 and are labeled with their ΔG values of 9.3, 6.9, and 4.3 kcal/mol. For reference, green-colored hydration sites have ΔG values closer to zero. (b) WaterMap hydration sites for the PRDM9/SAM/MRK-740 complex. The hydration sites observed with SAM are overall very similar to those observed with the SAH-based complex, with the notable exception that the three high-energy hydration sites identified in the PRDM9/SAH/MRK-740 complex are absent.

a

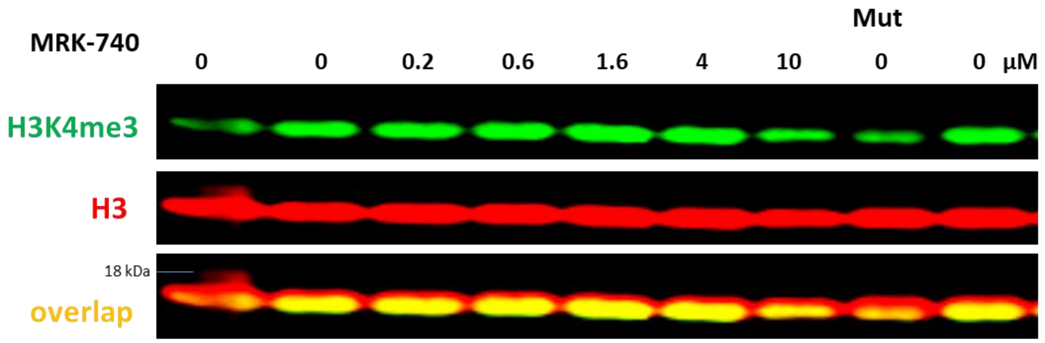

b

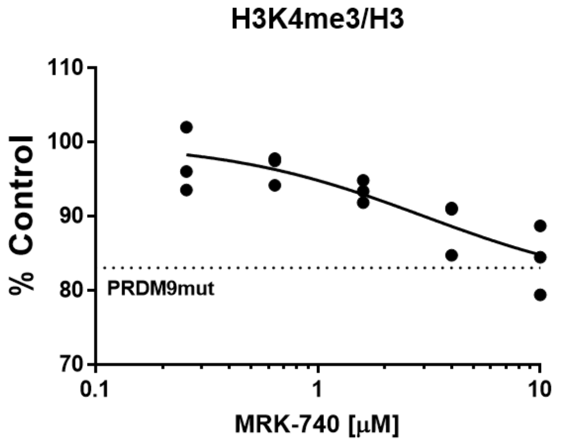

Supplementary Figure 9. MRK-740 inhibits PRDM9-dependent lysine trimethylation of endogenous histone H3. (a) Western blot analysis of cellular lysates prepared from HEK293T cells transfected with PRDM9-FLAG and treated with the indicated concentration of compound for 20 h. Mut denotes PRDM9 catalytic mutant (Y357S). (b) Non-linear fit of H3K4me3 fluorescence intensities normalized to intensities of histone H3 is shown. The results are MEAN +/- SEM of n=3 (technical replicates). Source data are provided as a Source Data file.

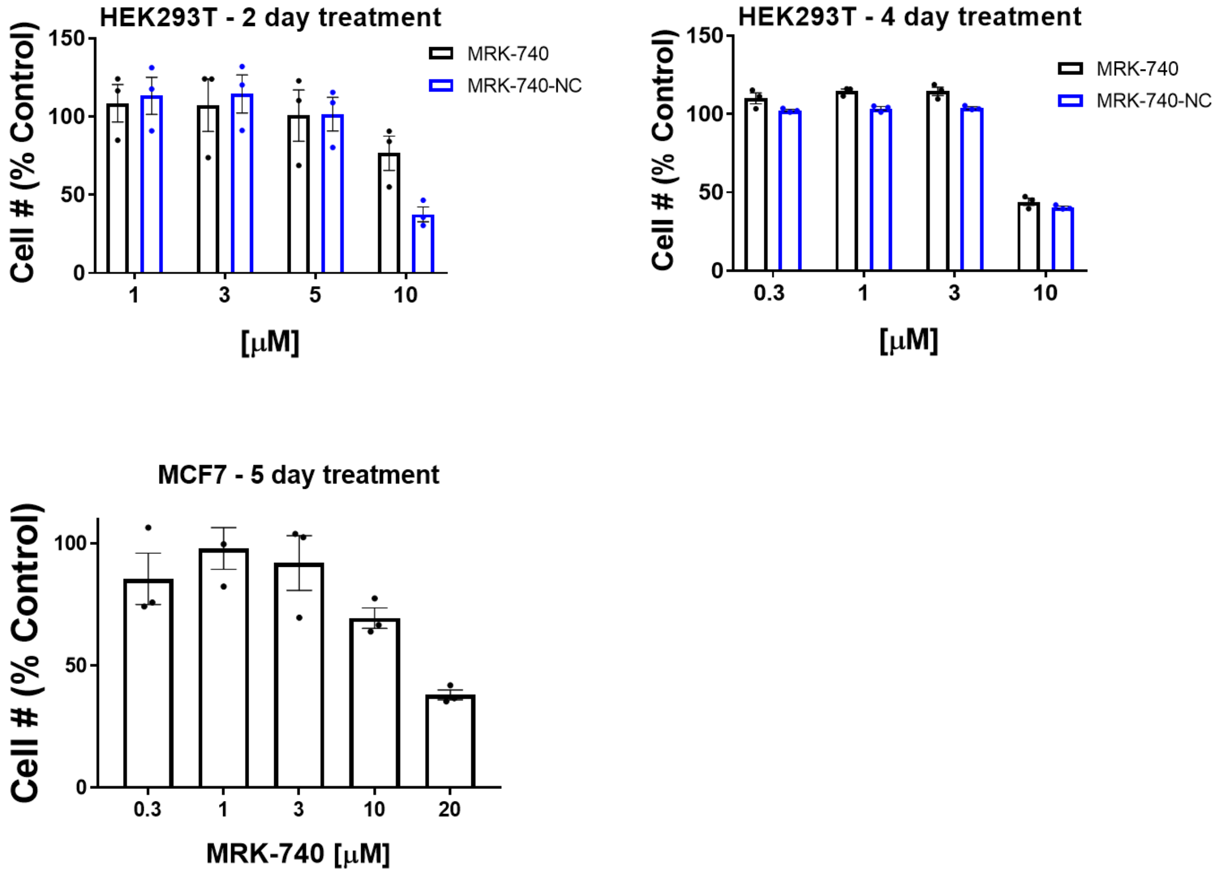

Supplementary Figure 10. The effect of MRK-740 and MRK-740-NC on HEK293 cell growth (top graphs) and MCF7 cells (bottom graph). Cells were treated with compounds for 2 and 4 days or 5 days in case of MCF7. Cell number was measured using IncuCyte™ ZOOM live cell imaging device. The results are MEAN +/- SEM, n=3 (technical replicates). Source data are provided as a Source Data file.

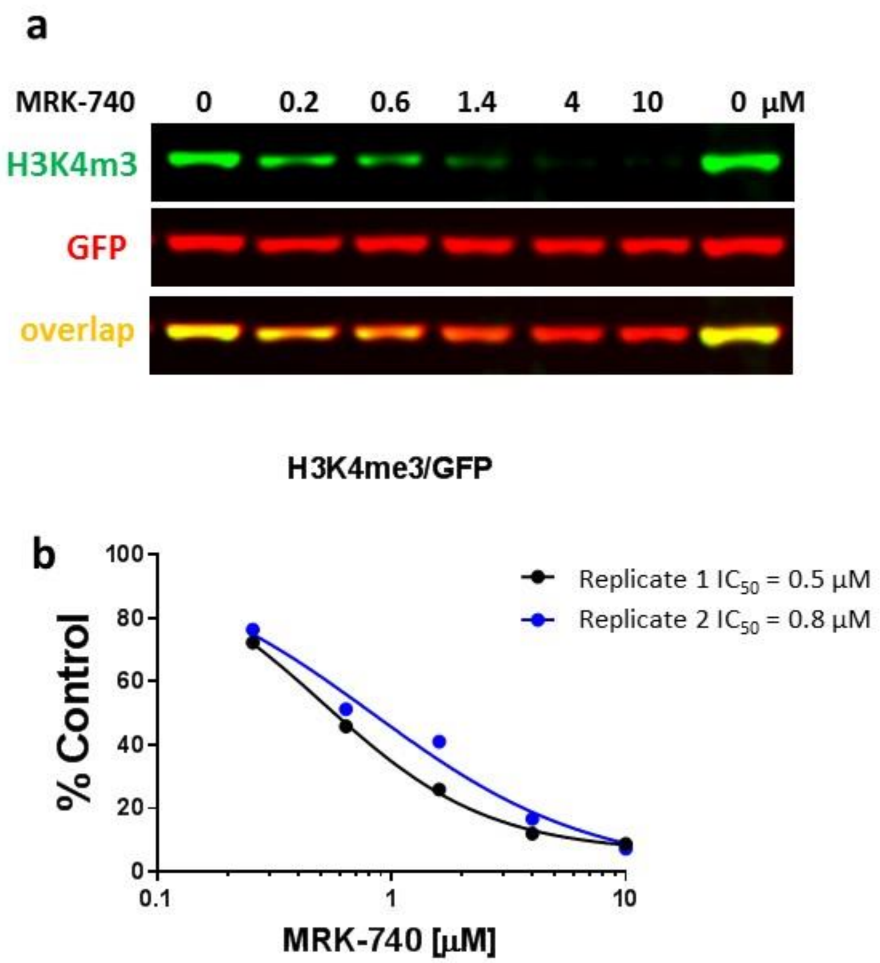

Supplementary Figure 11. MRK-740 inhibits PRDM9-dependent lysine trimethylation of exogenous histone H3 in MCF7 cells. (a) Western blots indicate MRK-740 decreases PRDM9-dependent K4 trimethylation of exogenous histone H3 in MCF7 cells. Cells were co-transfected with H3-GFP and PRDM9-FLAG and treated with compounds at indicated concentrations for 20 h. (b) The graph represents non-linear fit of H3K4me3 fluorescence intensities normalized to intensities of GFP. The results are from two independent experiments (n=2). Source data are provided as a Source Data file.

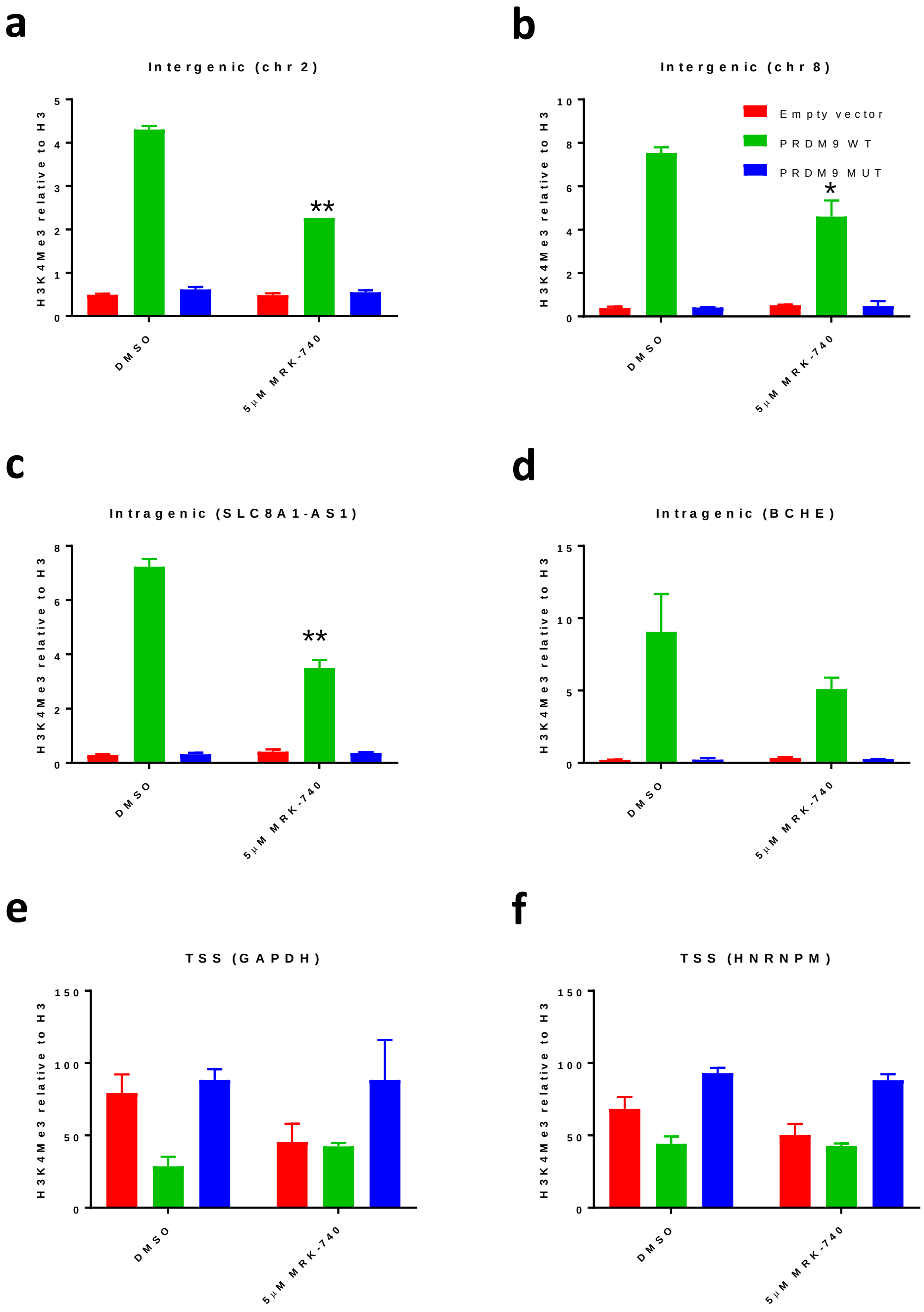

Supplementary Figure 12. MRK-740 selectively depletes PRDM9-dependent H3K4me3 formation. ChIP qPCR analyses of H3K4me3 methylation levels at known PRDM9-bound and control loci. Cells transfected with empty vector (negative control) or vectors overexpressing either wild-type PRDM9 (PRDM9 WT) or catalytically dead PRDM9 (PRDM9 MUT) were treated with DMSO or 5 μM MRK-740 for 48 hours. Panels a-d represent reported loci of

PRDM9 methylation; e-f are TSSs which are not known sites of PRDM9 methylation. Data are normalized to total H3 and are presented as the mean ± upper and lower limits from two replicates. Representative plots of two independent ChIP-qPCR experiments are shown. The chromosome coordinates and the genomic features associated with each of the assayed loci are indicated above each plot. Left tailed Student T-Tests were performed by comparing DMSO treated cells to compound treated cells that have been transfected with the same plasmids. *: p-value <0.05 and ** : p-value<0.01. Here we show a representative of two independent experiments (Mean+/- s.d of technical replicates) for our ChIP-qPCR experiments. Data obtained using this technique are inherently noisy due to stochastic differences in gene activity/histone positioning. Data are thus typically presented as representative rather than averaged. Source data are provided as a Source Data file.

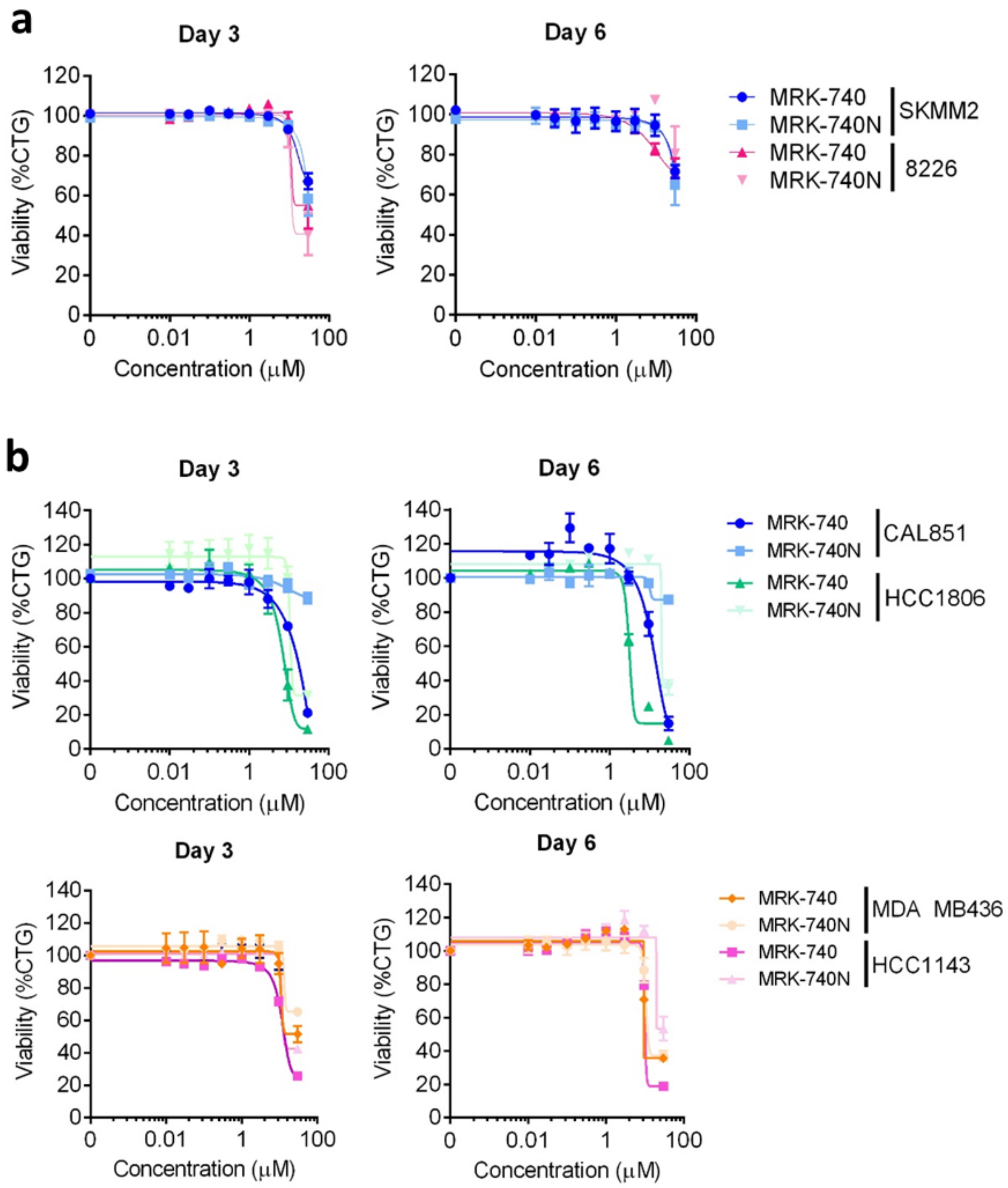

Supplementary Figure 13. Effect of MRK-740 on viability of cancer cell lines. Viability of (a) Multiple Myeloma lines and (b) Breast Cancer cell lines following 3 or 6 days treatment with MRK-740 and MRK-740-NC were assessed using CelltiterGlo (Promega) following manufacturer's instructions as described in the Supplementary methods. Data are normalized to DMSO control. N=3 biological replicates, +/- SEM. Source data are provided as a Source Data file.

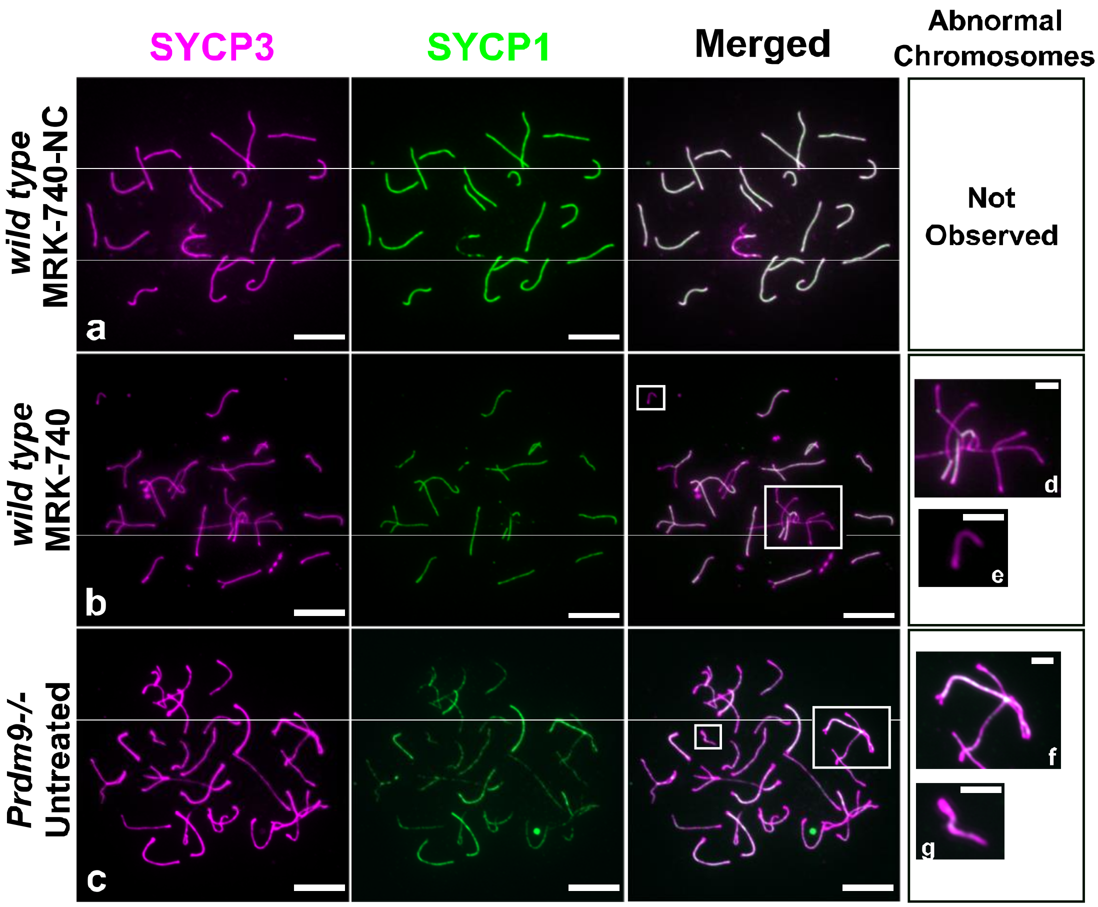

Supplementary Figure 14. Comparison of meiotic progression in the presence of MRK-740-NC and MRK-740 treated spermatocytes. Representative images of meiotic chromosome spreads prepared from p15 wild type mice (a) treated with MRK-740-NC or (b) MRK-740, and (c) untreated Prdm9-/- mice are presented. At p12, mice were treated with either MRK-740-NC or MRK-740 or received no treatment. Immunofluoresence co-staining was performed with SYCP1 (green) and SYCP3 (magenta) antibodies to visualize the formation of the synaptonemal complex during pachytene stage of meiosis I. Normal synaptonemal complex formation determined by full co- localization of SYCP1 and SYCP3 on autosomes was seen in wild-type mice treated with MRK-740-NC. No abnormalities in the synaptonemal complex were seen in

these mice and the morphology of pachytene stage spermatocytes was identical to that seen in wild-type mice receiving no treatment (not shown). Abnormal synaptonemal complex formation such as non-homologous synapsis and incomplete synapsis of autosomes was observed in wild-type mice treated with MRK-740 (b, d and e) in a similar manner seen in Prdm9-/- mice receiving no treatment (c, f and g). Scale bars are representative of (5µm) in a-c and (1.25µm) in d-g.

Supplementary Methods

Methyltransferase selectivity assay[1]

The effect of chemical probes and negative controls on the methyltransferase activities of protein, DNA and RNA methyltransferases was tested by radiometric assays using ^{3}H-SAM. For proteins such as MLL1 trimeric, MLL3 pentameric, EZH1 (PRC2) pentameric, and EZH2 (PRC2) trimeric complexes, as well as G9a, GLP, SUV39H1, SUV39H2, SUV420H1, SUV420H2, SETD2, SETD8, SETDB1, SETD7, PRMT1, PRMT3, PRMT4, PRMT5/ MEP50 complex, PRMT6, PRMT7, PRMT8, PRMT9, PRDM9, SMYD2, SMYD3, DNMT1, and BCDIN3D the incorporation of a tritium-labeled methyl group into biotinylated substrate was monitored using scintillation proximity assay (SPA). Briefly, a 10 µL reaction containing ^{3}H-SAM and substrate at concentrations close to the apparent K_m values for each enzyme (balanced conditions) was prepared. The reactions were quenched with 10 µL of 7.5 M guanidine hydrochloride; 180 µL of 20 mM Tris buffer (pH 8.0) were added, and the mixture was transferred to a 96-well FlashPlate and incubated for 1 h. The counts per minute (CPM) was measured on a TopCount plate reader. The CPM in the absence of compound or enzyme was defined as 100% activity and background (0%), respectively, for each dataset. For DNMT1, the double-stranded DNA substrate was prepared by annealing two complementary strands (biotinylated forward strand: B-GAGCCCGTAAGCCCGTTCAGGTCG and reverse strand: CGACCTGAACGGGCTTACGGGCTC) that were synthesized by Eurofins MWG Operon (Louisville, KY, USA).

For proteins which were tested with nucleosome as substrate such as DOT1L, NSD1, NSD2, NSD3, and ASH1L, or unbiotinylated Poly(2′-deoxyinosinic-2′-deoxycytidylic acid) (Cat# 81349-500UG, Sigma Aldrich) such as DNMT3A/3L, and DNMT3B/3L, a filter-based assay was used. In this assay, a trichloroacetic acid (TCA) protein precipitation protocol was employed. A 10 µL reaction mixture was incubated at 23°C for 1 h, followed by addition of 50 µL of 10% TCA. The mixture was transferred to filter plates (Millipore, Billerica, MA, USA) that were centrifuged at 2000 rpm (Allegra X-15R; Beckman Coulter, Brea, CA, USA) for 2 min. Samples were washed twice with 10% TCA and once with ethanol (180 µL), and centrifuged. After drying, 100 µL MicroScint-O (Perkin Elmer) was added to each well and the plates were centrifuged to remove the liquid. A 70 µL volume of MicroScint-O was added and the CPM was measured with a TopCount plate reader.

CelltiterGlo viability cell assay

Cells were seeded in 96 well plates at per-well densities of 10000 (MDA-MB436, ATCC-HTB130), 15000 (8226, kind gift from Rodger Tiedemann, Princess Margaret Cancer Centre), 20000 (HCC1806, ATCC-CRL 2335), 20000 (HCC1143, ATCC-CRL2321), 20000 (SKMM2, kind gift from Rodger Tiedemann, Princess Margaret Cancer Centre) and 50000 (CAL851, kind gift from Mathieu Lupien, Princess Margaret Cancer Centre) and were treated at increasing concentrations of compounds ranging from 0.01 μM to 30 μM and assessed for proliferation using CelltiterGlo (Promega) following manufacturer's instructions, as readout on days 3 and 6 as indicated. All cell lines were mycoplasma negative, as determined by MycoAlert™ Mycoplasma Detection Kit(Lonza).

Transgenic mouse lines used in this study

Prdm9KO [2], mice have been described and were maintained on a C57BL/6 background. Mice were housed under standard conditions, were maintained on a 12- hour light/dark cycle, were fed a standard chow diet containing 6% crude fat, and were treated in compliance with the institutional guidelines for animal care and use. All experimental protocols were approved by the Animal Care and Use Committee of Biological Resource Centre at Biopolis, A*STAR, Singapore (protocol#171268).

Testis microinjection protocol

Seminiferous tubule microinjection was performed as previously described [3,4]. At postnatal day 12 (P12) A small incision was made in the abdomen of the mouse and testes were exposed by pulling the abdominal fat pad. Testis were injected using a micro-capillary pipette injector (made in-house). Surgical incisions were closed using 12mm Silkam sutures (Braun; 762075). 2 µl of either 1 µM MRK-740-NC or 1 µM MRK-740 compounds were administered into both testis of injected mice for each experiment. To make working dilutions of these drugs 100% ethanol was first used to dissolve the initial lyophilized drug and then this solution was further diluted in PBS to the desired concentration. Injected mice were left to recover for three days until they were

sacrificed at P15 for meiotic chromosome spread preparation. No pharmacokinetic data was generated in these experiments.

Preparation of cell solution from mouse testes for meiotic chromosome spreads: drying down methodology

P15 mice were euthanized and testes were surgically excised and placed into PBS. Upon excision the tunica albuginea was removed from each testis and discarded. From here the seminiferous tubules of both testes were combined and lightly pulled apart using forceps in a solution of 2.2% trisodium citrate solution (75 mM). Seminiferous tubules were then moved into a hypotonic solution buffer (30 mM Tris-HCl pH8.8, 5 mM EDTA, 17 mM Trisodium citrate dihydrate, 50 mM Sucrose) for 20 minutes. After 20 minutes, seminiferous tubules were moved into a solution of 100mM Sucrose solution pH8.2. Here tubules were chopped finely with a razorblade in a petri dish until they could be easily pipetted through an uncut 200µL pipette tip. Cells were flushed from cut tubules by repeatedly passing tubules through a 200 µL tip (30X). The now cloudy sucrose solution was then separated from the cut tubules by tilting the petri dish to allow liquid to pool at the edges of the petri dish. This solution was then be collected and passed through a 100µm nylon mesh cell strainer (2X). This solution was used for downstream cell spreading.

Preparation of fixative and coating of microscope slides: 500mg of paraformaldehyde was dissolved in 50 mL of PBS (1% final w/v) containing 0.33% Triton X-100 adjusted to pH9.2. Superfrost Plus Microscope slides (Thermofisher #4951PLUS4) were immersed in the fixative solution to coat the surface of the slide.

Cell spreading procedure: A single drop of cell solution ~30 µL (as prepared above) was dropped from a height of approximately 5cm onto one of the top corners of a fixative coated slide whilst the slide was held at a slight downward angle. Via gentle tilting of the slide the cell solution was guided to coat the entire surface of the slide. When the cell solution had fully coated the slide, the slide was held flat and the slide was humidified by blowing onto the slide 5-10X until newton’s rings (iridescent rings) were briefly seen. The slide was then placed into a humidified box kept at 40-50ºC for at least 2hrs (maximally 16 hours). After this incubation period the lid to the humidified box was removed and slides were allowed to partially dry by fanning for

approximately 1-2 minutes on a benchtop. Slides were then moved into -80ºC for (indefinite) storage until staining.

Immunofluorescent staining of meiotic chromosome spreads

Blocking: Slides for immunostaining were washed in water containing 0.1% dishwashing liquid for 5 minutes (1X) and subsequently in PBS for 5 minutes (3X). Blocking solution [PBS containing 10% BSA (Sigma Aldrich #A7906), 3% milk powder (Bio-Rad #1706404), 0.15% Triton X-100], was applied to slides for 1 hour at 4 ºC, under constant rocking. Blocking solution was held on slides by drawing a hydrophobic barrier using a DAKO marker (Agilent, # S2002).

Primary antibody incubation: Anti-SYCP3 (Santa Cruz, #Sc-20845) and anti-SYCP1 antibodies (Abcam #Ab15087) were each diluted 1/150 in blocking solution (as above). Primary antibodies were applied to slides for 16 hours at 4 ºC, under constant rocking. Secondary antibody incubation: Donkey anti-goat Alexa-Fluor-555 (Thermofisher #A21432) and Donkey anti-mouse Alexa-Fluor-488 (Thermofisher #A21202) were each diluted 1/500 in blocking solution (as above). Secondary antibodies were applied to slides for 1 hour at 4 ºC, under constant rocking.

Washing: After blocking and primary and secondary antibody incubation steps, slides were washed for 5 minutes in PBST (PBS containing 0.5% Tween20) (3X).

Coverslip placement and imaging: Following immunostaining, Slides were washed for 5 minutes in PBS. Immu-mount aqueous immersion (Thermo-scientific #9990402) was then applied to slides. Coverslips were applied and sealed with nail polish.

Microscope image acquisition

Fluorescence imaging: All fluorescence microscope images were taken using a Zeiss AxioImager Z1 (EBL) motorized microscope. 100X magnification images were taken using an oil immersion Plan Apochromat lens with a 1.4 numerical aperture. All images were taken at room temperature using Immersol immersion oil as the imaging medium. Specific signal of primary antibodies was detected using Alexa-Fluor secondary antibodies conjugated to either Alexa-Fluor 488 or 500 fluorophore dyes. Images were taken using an Axio cam Hrc camera using X-cite metal halide as a fluorescence source. Images were acquired using the Zen 2.3 (blue edition) acquisition software. After imaging further processing was performed using Adobe Photoshop CC 2018 to add pseudo colours and to overlay different channels of co-stained

images. Where comparisons were drawn between fluorescent images lamp intensity and exposure time was kept identical when taking images.

Primary antibodies used for immunofluorescence of meiotic spreads						
**Protein**	**Species**	**Company**	**Cat #**	**Used for**	**Dilution**	**Diluted in**
Sycp1	Rabbit (polyclonal)	Abcam	15087	Immunofluorescence (Meiotic spreads)	150	Immunofluorescence blocking buffer (10% BSA, 3% milk powder, 0.15% Triton X in PBS)
Sycp3	Goat (Polyclonal)	Santa-Cruz	20845	Immunofluorescence (Meiotic spreads)	150	Immunofluorescence blocking buffer (10% BSA, 3% milk powder, 0.15% Triton X in PBS)

Antibody	Company	Cat #	Used for	Dilution	Diluted in
Donkey Anti-Goat 555 (Alexa-fluor)	Thermofisher	A21432	Detection of Immunofluorescence	500	Immunofluorescence blocking buffer (10% BSA, 3% milk powder, 0.15% Triton X in PBS)
Donkey Anti-Mouse 488 (Alexa-fluor)	Thermofisher	A21202	Detection of Immunofluorescence	500	Immunofluorescence blocking buffer (10% BSA, 3% milk powder, 0.15% Triton X in PBS)

Chemical Synthesis

Synthesis of 3-(3,5-dimethoxyphenyl)-5-(1-methyl-9-(2-methylpyridin-4-yl)-1,4,9-triazaspiro[5.5]undecan-4-yl)-1,2,4-oxadiazole (MRK-740)

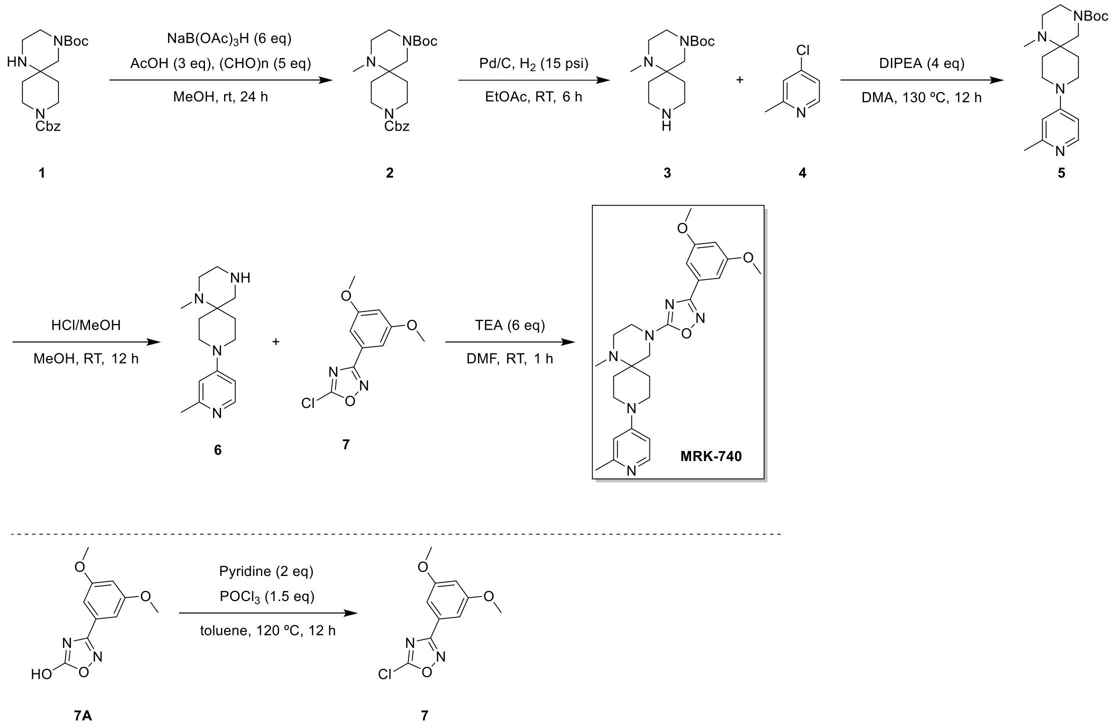

Synthesis of 9-benzyl 4-tert-butyl 1-methyl-1,4,9-triazaspiro[5.5]undecane-4,9-dicarboxylate (2)

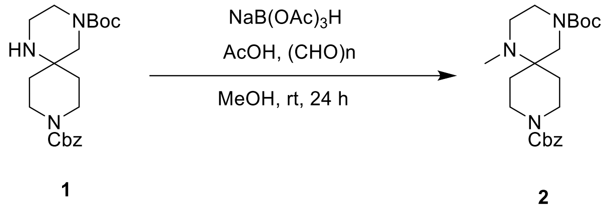

To a solution of 9-benzyl 4-tert-butyl 1,4,9-triazaspiro[5.5]undecane-4,9-dicarboxylate (6.4 g, 16.43 mmol), paraformaldehyde (7.40 g, 82 mmol) in MeOH (120 mL) was added acetic acid (2.068 mL, 36.1 mmol). The reaction mixture was stirred at 25 ^{o}C for 20 min and sodium triacetoxyborohydride (13 g, 61.3 mmol) was added in portions. The reaction mixture was stirred for 3 h after which additional sodium triacetoxyborohydride (13 g, 61.3 mmol) was added in portions and the reaction mixture was stirred for another 8 h. The reaction mixture was quenched with H_2O (300 mL) and $NaHCO_3$ (saturated aq.) was added until the pH reached 7-8. The

aqueous layer was extracted with dichloromethane (3 X 200 mL) and the combined organic layers were washed with brine (500 mL), dried over anhydrous $MgSO_4$ and filtered. The filtrate was concentrated under reduced pressure. The crude product was purified by flash silica gel chromatography (ISCO; 12 g Agela Silica Flash Column, eluent of 0~5% MeOH/DCM gradient @ 30 mL.min^{-1}) to give 9-benzyl 4-tert-butyl 1-methyl-1,4,9-triazaspiro[5.5]undecane-4,9-dicarboxylate (5.9 g, 89% yield) as a colorless oil.

LCMS (ESI) calc'd for $C_{22}H_{34}N_3O_4$ $[M+H]^+$: 404.3, found: 404.2.

Synthesis of tert-butyl 1-methyl-1,4,9-triazaspiro[5.5]undecane-4-carboxylate (3)

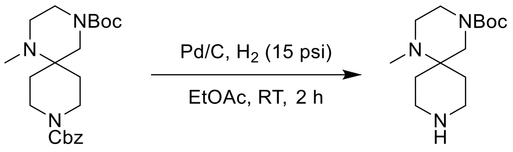

2 3

9-benzyl 4-tert-butyl 1-methyl-1,4,9-triazaspiro[5.5]undecane-4,9-dicarboxylate (5.9 g, 14.62 mmol) was added to a mixture of Pd/C (1.556 g, 1.462 mmol) (10%, wet) in EtOAc (150 mL). The resulting suspension was degassed and backfilled with H_2 three times, and then stirred at 25 ^oC under H_2 at 15 psi (hydrogen balloon) for 2 h. The mixture was filtered and solvent was evaporated to give tert-butyl 1-methyl-1,4,9-triazaspiro[5.5]undecane-4-carboxylate (3.91 g, 99% yield) as a colorless oil which was used in the next step without purification.

Synthesis of tert-butyl 1-methyl-9-(2-methylpyridin-4-yl)-1,4,9-triazaspiro[5.5]undecane-4-carboxylate (5)

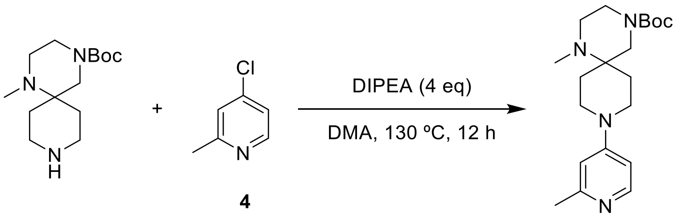

3 4 5

tert-butyl 1-methyl-1,4,9-triazaspiro[5.5]undecane-4-carboxylate (2.7 g, 10.02 mmol) was added to a mixture of 4-chloro-2-methylpyridine hydrochloride (3.29 g, 20.05 mmol) and DIPEA (7.00 mL, 40.1 mmol) in DMA (30 mL). The resulting suspension was degassed and backfilled with N_2 for three times, and then stirred at 130 °C for 13 h. The mixture was cooled to 23 °C, water (200 mL) was added and the mixture was extracted with DCM (3 x 100 mL). The combined organic fractions were washed with brine (saturated, 300 mL), dried (anhydrous Na_2SO_4), filtered and the solvent evaporated under reduced pressure. The crude product was purified by flash silica gel chromatography (ISCO; 12 g Agela Silica Flash Column, Eluent of 0~7% MeOH/DCM gradient @ 30 mL.min^{-1}) to give tert-butyl 1-methyl-9-(2-methylpyridin-4-yl)-1,4,9-triazaspiro[5.5]undecane-4-carboxylate (2.1 g, 58% yield) as a colorless oil.

LCMS (ESI) calc'd for $C_{20}H_{33}N_4O_2$ $[M+H]^+$: 361.3, found: 361.1

Synthesis of 5-chloro-3-(3,5-dimethoxyphenyl)-1,2,4-oxadiazole (7)

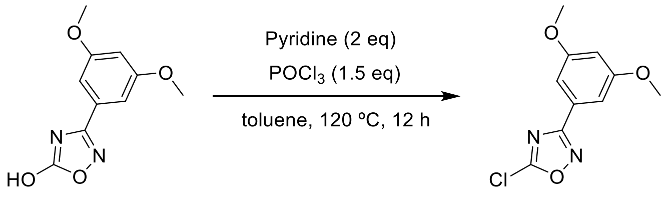

7A **7**

$POCl_3$ (2.077 mL, 22.28 mmol) was added dropwise to a stirred mixture of 3-(3,5-dimethoxyphenyl)-1,2,4-oxadiazol-5(4*H*)-one (3.3 g, 14.85 mmol), pyridine (2.402 mL, 29.7 mmol) in toluene (80 mL) at 0-5 °C and the mixture was stirred at 120 °C for 8 h.. The mixture was cooled to 23 °C, and the solvent was evaporated under reduced pressure to give 5-chloro-3-(3,5-dimethoxyphenyl)-1,2,4-oxadiazole (3.1 g) as a yellow solid which was used in the next step without purification.

Synthesis of 1-methyl-9-(2-methylpyridin-4-yl)-1,4,9-triazaspiro[5.5]undecane dihydrochloride

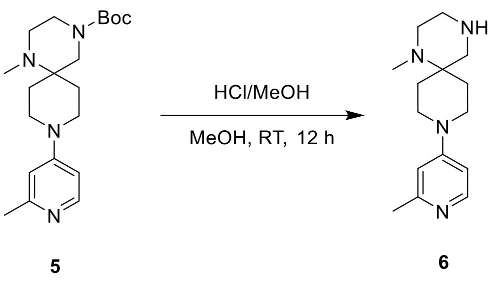

HCl (20 mL, 80 mmol, 4 M in MeOH) was added to a mixture of tert-butyl 1-methyl-9-(2-methylpyridin-4-yl)-1,4,9-triazaspiro[5.5]undecane-4-carboxylate (1.8 g, 3.99 mmol) in MeOH (10 mL). The resulting suspension was stirred at 45 °C for 40 min. The mixture was evaporated under reduced pressure to give 1-methyl-9-(2-methylpyridin-4-yl)-1,4,9-triazaspiro[5.5]undecane, 2HCl (1.6 g) as a yellow solid which was used in the next step without purification.

Synthesis of 3-(3,5-dimethoxyphenyl)-5-(1-methyl-9-(2-methylpyridin-4-yl)-1,4,9-triazaspiro[5.5]undecan-4-yl)-1,2,4-oxadiazole (MRK-740)

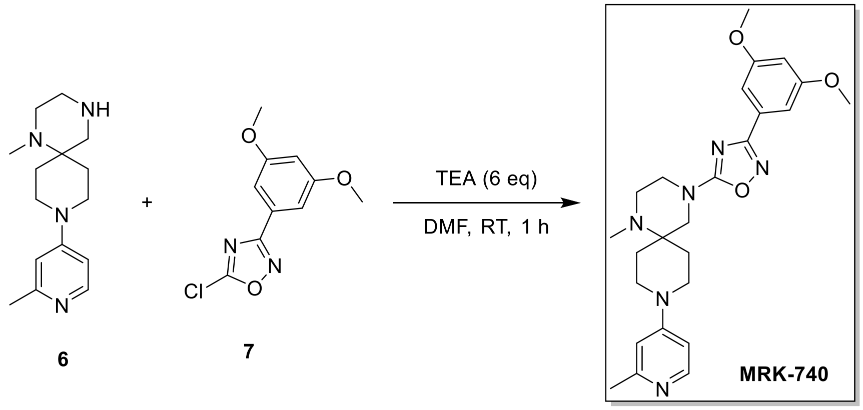

Triethylamine (5.85 mL, 42.0 mmol) was added to a stirred mixture of 1-methyl-9-(2-methylpyridin-4-yl)-1,4,9-triazaspiro[5.5]undecane dihydrochloride (1.4 g, 4.20 mmol) and 5-chloro-3-(3,5-dimethoxyphenyl)-1,2,4-oxadiazole (2.02 g, 8.40 mmol) in DMF (20 mL) and the mixture was stirred at 0 °C for 30 min. Water (100 mL) and aq. HCl (4 M) was added to adjust the pH to 3, and the mixture was extracted with ethyl acetate (3 x 30 mL). NH_4OH was added to

the water phase to adjust the pH to 10, then the mixture was extracted with DCM (3 x 30 mL). The combined organic fractions were washed with brine (saturated, 100 mL), dried (Na_2SO_4), and evaporated. The residue was purified by prep-HPLC (Column Waters Xbridge Prep OBD C18 100*19mm*5um Conditions: water (0.04% $NH_3 \cdot H_2O$+10 mM NH_4HCO_3) - MeCN beginning at 36% MeCN and ending at a 66% MeCN, gradient over 10 minutes followed by 2 mins at 100 % MeCN with a flow rate of 25 $mL.min^{-1}$) to give 3-(3,5-dimethoxyphenyl)-5-(1-methyl-9-(2-methylpyridin-4-yl)-1,4,9-triazaspiro[5.5]undecan-4-yl)-1,2,4-oxadiazole (1 g, 2.110 mmol) as a brownish solid. The compound was additionally purified by SFC (Basic, 21 x 250 (mm), 30%/70% Methanol/CO2 + 0.1% NH_4OH) and lyophilized to afford 3-(3,5-dimethoxyphenyl)-5-(1-methyl-9-(2-methylpyridin-4-yl)-1,4,9-triazaspiro[5.5]undecan-4-yl)-1,2,4-oxadiazole (852 mg; 44% yield) as a pale-yellow solid**.** **LCMS (ESI)** calc'd for $C_{25}H_{33}N_6O_3$ $[M+H]^+$: 465.2 , found: 465.2; **^{1}H NMR** (400MHz, CD_3OD) δ 7.98 (d, *J* = 6.2 Hz, 1H), 7.08 (d, *J* = 2.3 Hz, 2H), 6.68 (d, *J* = 2.6 Hz, 1H), 6.64 (dd, *J* = 6.3, 2.6 Hz, 1H), 6.61 (t, *J* = 2.3 Hz, 1H), 3.26 (ddd, *J* = 13.6, 10.7, 3.3 Hz, 2H), 2.87 – 2.83 (m, 2H), 2.38 (s, 3H), 2.38 (s, 3H), 1.98 (ddd, *J* = 14.8, 10.8, 4.6 Hz, 2H), 1.66 (dd, *J* = 13.9, 3.9 Hz, 2H)

Synthetic Scheme for MRK-740 NC

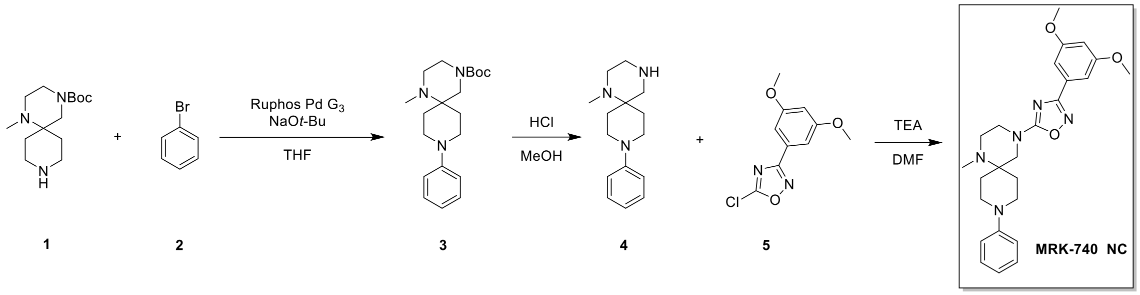

1 2 3 4 5

Preparation of MRK-740-NC

Preparation of 3

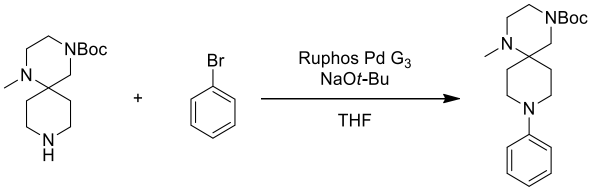

1 2 3

To a solution of tert-butyl 1-methyl-1,4,9-triazaspiro[5.5]undecane-4-carboxylate (800 mg, 2.97 mmol) in THF (2 ml) was added bromobenzene (932 mg, 5.94 mmol), sodium t-butoxide (570 mg, 5.94 mmol) and methanesulfonato(2-dicyclohexylphosphino-2',6'-di-i-propoxy-1,1'-biphenyl)(2'-amino-1,1'-biphenyl-2-yl)palladium(II) (248 mg, 0.296 mmol). The mixture was stirred at 80 °C for 17 h. The mixture was filtered through Celite and purified by prep-TLC (SiO_2, DCM: MeOH=10:1) to give tert-butyl 1-methyl-9-phenyl-1,4,9-triazaspiro[5.5]undecane-4-carboxylate (600 mg, 50% yield) as a colorless oil. **MS (ESI)** calcd. for $C_{20}H_{32}N_3O_2$ $[M+H]^+$, 346.5, found, 346.2

Preparation of 4

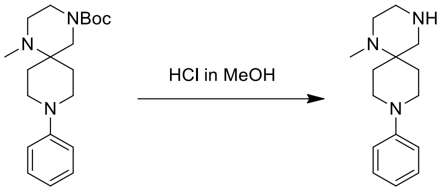

3 4

To tert-butyl 1-methyl-9-phenyl-1,4,9-triazaspiro[5.5]undecane-4-carboxylate (400 mg, 1.158 mmol) was added HCl in MeOH (0.289 mL, 1.158 mmol, 4 M). The mixture was stirred at 20 °C for 2 h. The mixture was concentrated to give 1-methyl-9-phenyl-1,4,9-triazaspiro[5.5]undecane hydrochloride (320 mg, 78% yield) as a colorless solid.

MS (ESI) calcd. for $C_{15}H_{24}N_3$ ($M+H^+$) 246.4, found: 246.1

Preparation of MRK-740-NC

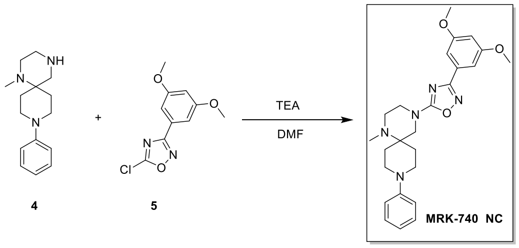

4 5

To a solution of 1-methyl-9-phenyl-1,4,9-triazaspiro[5.5]undecane hydrochloride (320 mg, 0.908 mmol) in DMF (2 mL) was added 5-chloro-3-(3,5-dimethoxyphenyl)-1,2,4-oxadiazole (219 mg, 0.908 mmol) and TEA (0.380 mL, 2.73 mmol). The mixture was stirred at 20 °C for 2 h.. The mixture was purified by prep-HPLC (basic) to give **MRK-740-NC** (103 mg, 98.76% purity, 25% yield) as a light yellow solid.

HPLC conditions: Column Xtimate C18 150x 25mm (5 µm). Conditions: water (10mM NH_4HCO_3) - MeCN beginning at 53% MeCN and ending at a 73% MeCN, gradient over 15 minutes followed by 2 mins at 100 % MeCN with a flow rate of 25 mL.min^{-1}.

MS (ESI) calcd. for $C_{25}H_{32}N_5O_3$ ($M+H^+$) 450.5, found: 450.2

^{1}H NMR (500 MHz, CD_3OD) δ ppm 7.20-7.26 (m, 2H), 7.07 (d, *J* = 2.44 Hz, 2H), 6.99 (d, *J* = 7.93 Hz, 2H), 6.84 (t, *J* = 7.25 Hz, 1H), 6.59 (t, *J* = 2.29 Hz, 1H), 3.69-3.86 (m, 10 H), 3.55 (brd, *J* = 12.66 Hz, 2H), 3.01 (brt, *J* = 10.99 Hz, 2H), 2.83-2.89 (m, 2H), 2.36-2.45 (m, 3H), 2.01-2.11 (m, 2H), 1.66 (brd, *J* = 13.28 Hz, 2 H).

Supplementary References

1. Scheer, S. et al. A chemical biology toolbox to study protein methyltransferases and epigenetic signaling. *Nat Commun* **10**, 19 (2019).
2. Hayashi, K., Yoshida, K. & Matsui, Y. A histone H3 methyltransferase controls epigenetic events required for meiotic prophase. *Nature* **438**, 374-8 (2005).
3. Kanatsu-Shinohara, M., Toyokuni, S. & Shinohara, T. Transgenic mice produced by retroviral transduction of male germ line stem cells in vivo. *Biol Reprod* **71**, 1202-7 (2004).
4. Ogawa, T., Arechaga, J.M., Avarbock, M.R. & Brinster, R.L. Transplantation of testis germinal cells into mouse seminiferous tubules. *Int J Dev Biol* **41**, 111-22 (1997).
