# Supplementary material for: Discovery of a chemical probe for PRDM9
Source: Nat Commun. 2019 Dec 17;10:5759. doi: 10.1038/s41467-019-13652-x (PMC6917776; doi:10.1038/s41467-019-13652-x)
Supplement: Supplementary file 3 — Description of Additional Supplementary Files [file 41467_2019_13652_MOESM3_ESM.docx]

**Description of Additional Supplementary Files**

**File Name: Supplementary Data 1**

**Description:** The chemical structures presented in Supplementary Figure 2 are provided in ChemDraw.

**File Name: Supplementary Data 2**

**Description:** The chemical structures presented in figures 1a and 1b are provided in ChemDraw.

**File Name: Supplementary Data 3**

**Description:** The compound NMR and MS quality control data are provided.

**File Name: Supplementary Data 4**

**Description:** Supplementary Table 2 is provided in excel format.
