# Supplementary material for: Discovery of a chemical probe for PRDM9
Source: Nat Commun. 2019 Dec 17;10:5759. doi: 10.1038/s41467-019-13652-x (PMC6917776; doi:10.1038/s41467-019-13652-x)

Extraction Ion Chromatograms at Proposed m/z

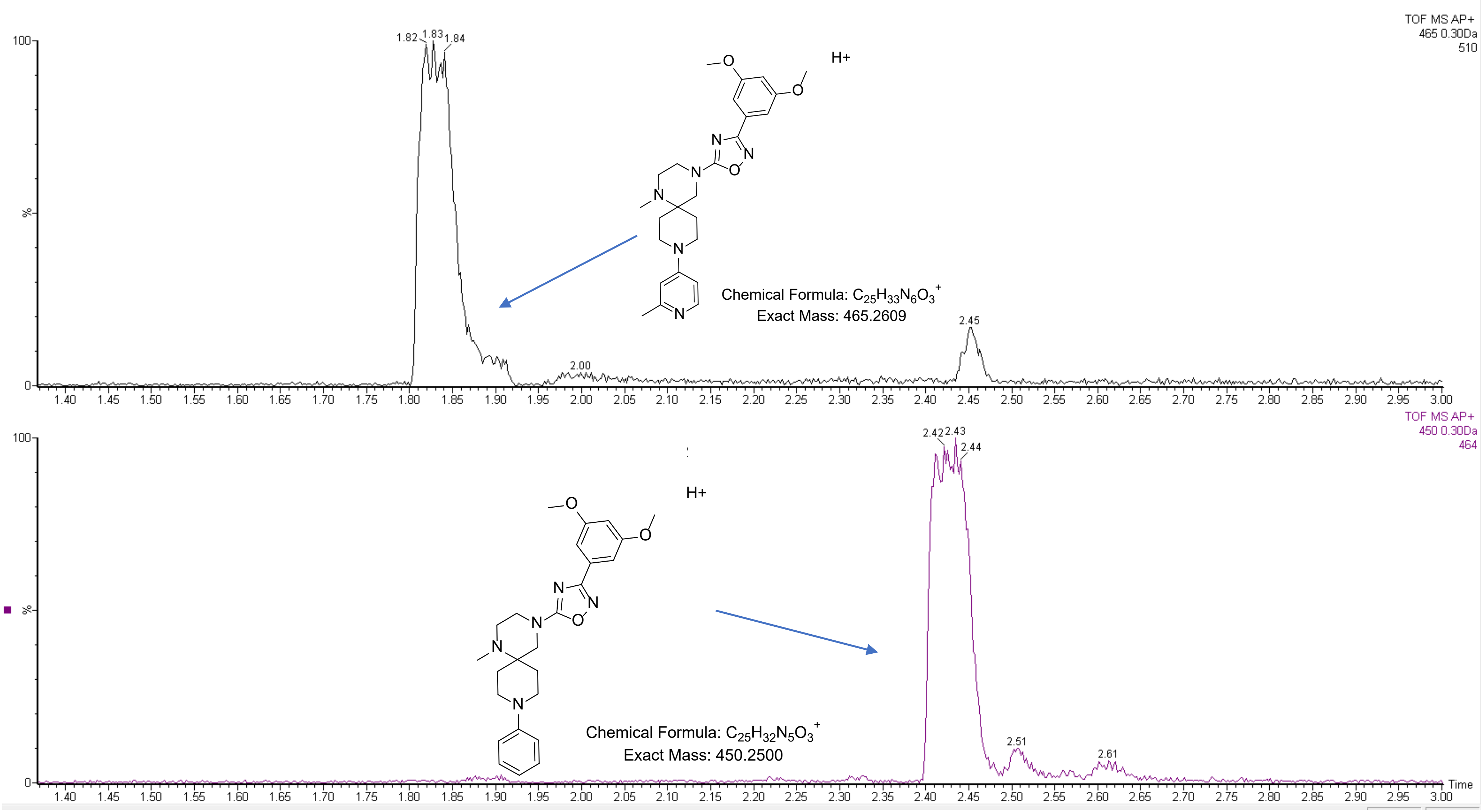

MRK-740

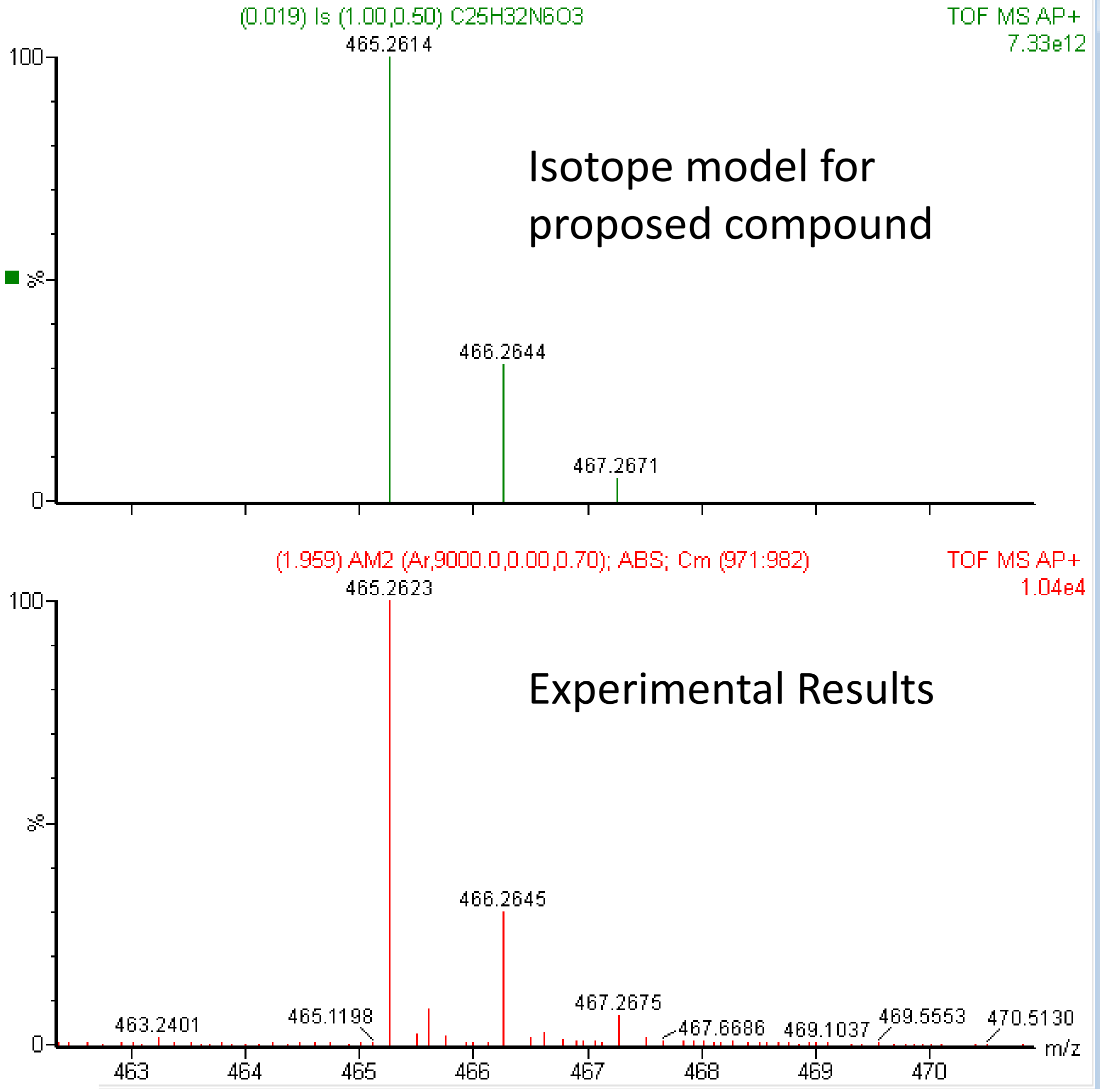

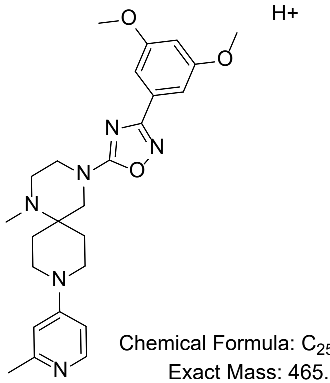

Chemical Formula: $C_{25}H_{33}N_6O_3^+$
Exact Mass: 465.2609

Single Mass Analysis
Tolerance = 20.0 PPM / DBE: min = -1.5, max = 50.0
Element prediction: Off
Number of isotope peaks used for i-FIT = 3
Monoisotopic Mass, Even Electron Ions
254 formula(e) evaluated with 8 results within limits (up to 50 closest results for each mass)
Elements Used:

Mass	Calc. Mass	mDa	PPM	DBE	Formula	i-FIT	i-FIT (Norm)	C	H	N	O	I
465.2623	465.2614	0.9	1.9	12.5	C25 H33 N6 O3	153.9	0.5	25	33	6	3	
	465.2641	-1.8	-3.9	11.5	C29 H37 O5	154.5	1.1	29	37		5	
	465.2654	-3.1	-6.7	16.5	C30 H33 N4 O	156.5	3.2	30	33	4	1	
	465.2574	4.9	10.5	8.5	C20 H33 N8 O5	157.5	4.1	20	33	8	5	
	465.2542	8.1	17.4	16.5	C31 H33 N2 O2	158.5	5.1	31	33	2	2	
	465.2593	3.0	6.4	0.5	C23 H46 O I	158.5	5.2	23	46		1	1
	465.2582	4.1	8.8	20.5	C36 H33	159.6	6.2	36	33			
	465.2706	-8.3	-17.8	0.5	C22 H46 N2 I	160.8	7.4	22	46	2		1

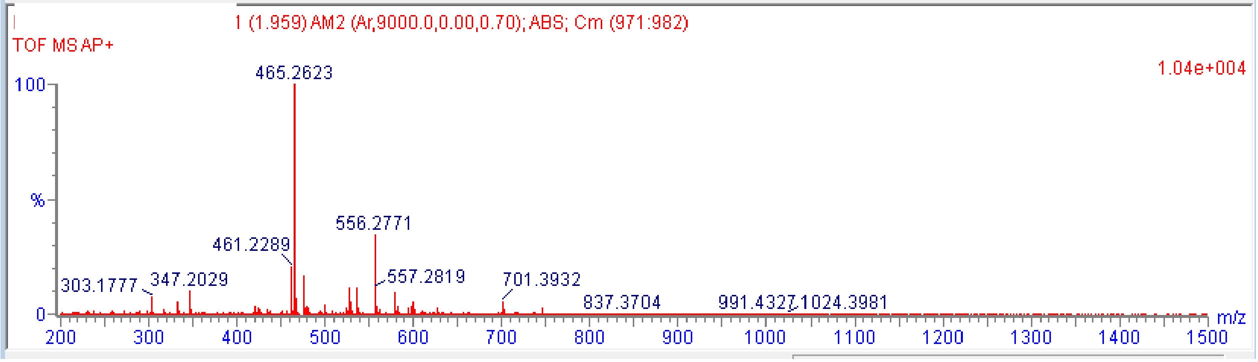

MRK740-NC

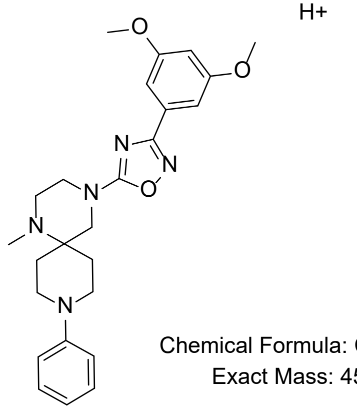

Chemical Formula: $C_{25}H_{32}N_5O_3^+$
Exact Mass: 450.2500

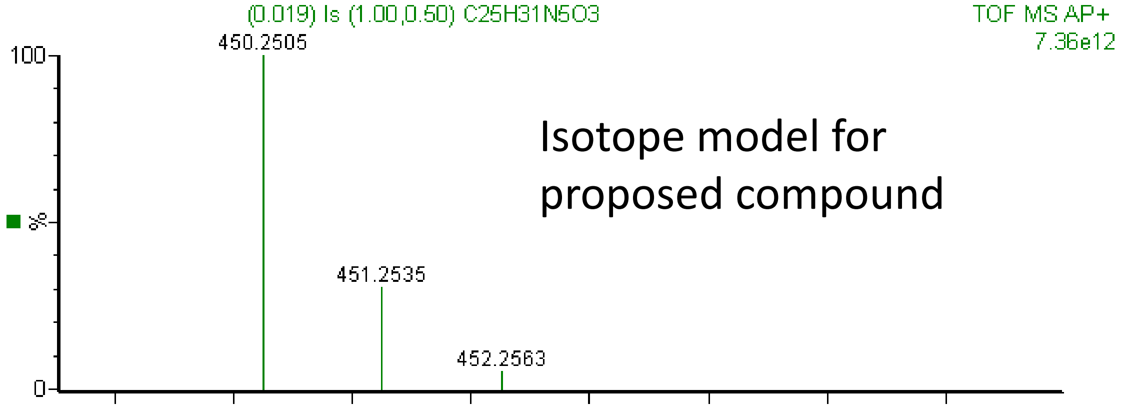

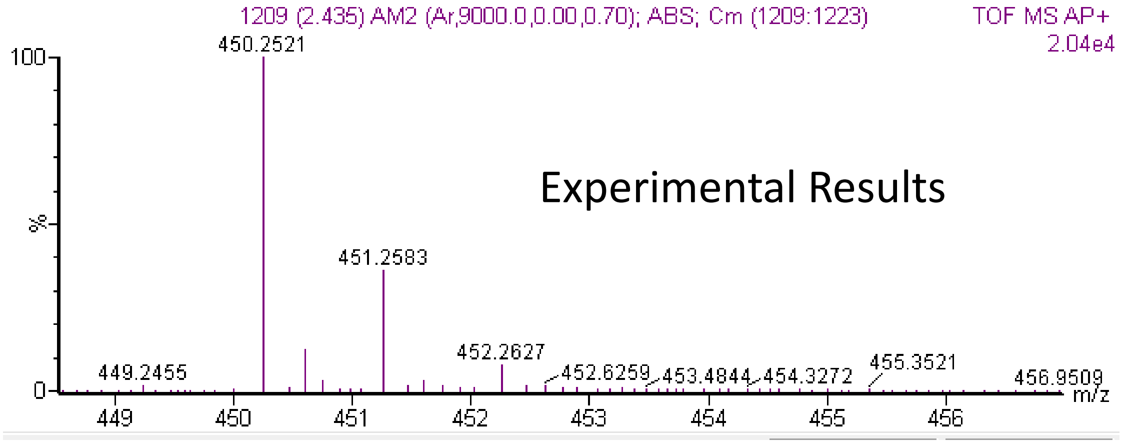

Single Mass Analysis
Tolerance = 20.0 PPM / DBE: min = -1.5, max = 50.0
Element prediction: Off
Number of isotope peaks used for i-FIT = 3
Monoisotopic Mass, Even Electron Ions
483 formula(e) evaluated with 6 results within limits (up to 50 closest results for each mass)
Elements Used:

Mass	Calc. Mass	mDa	PPM	DBE	Formula	i-FIT	i-FIT (Norm)	C	H	N	O	I
450.2521	450.2505	1.6	3.6	12.5	C25 H32 N5 O3	171.3	4.6	25	32	5	3	
	450.2545	-2.4	-5.3	16.5	C30 H32 N3 O	167.7	1.0	30	32	3	1	
	450.2465	5.6	12.4	8.5	C20 H32 N7 O5	173.8	7.1	20	32	7	5	
	450.2577	-5.6	-12.4	8.5	C19 H32 N9 O4	174.1	7.4	19	32	9	4	
	450.2597	-7.6	-16.9	0.5	C22 H45 N I	173.3	6.6	22	45	1		1
	450.2433	8.8	19.5	16.5	C31 H32 N O2	167.1	0.5	31	32	1	2	

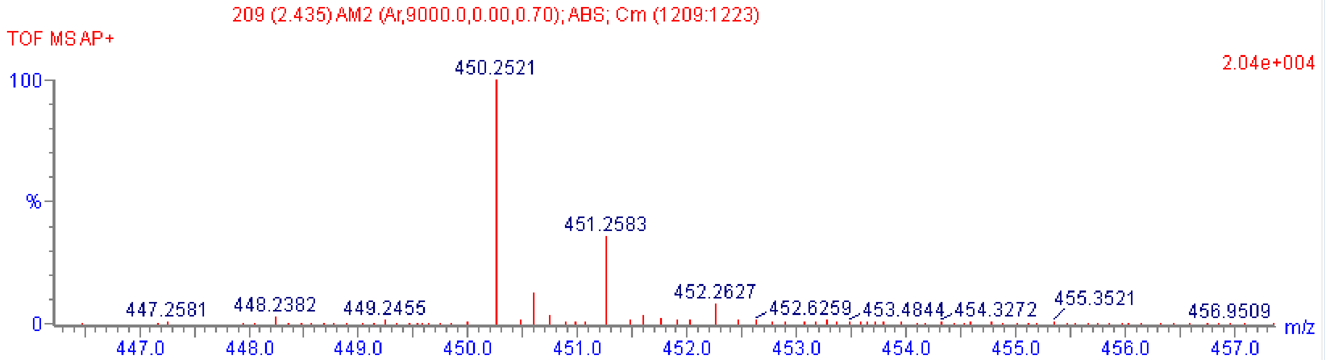

MRK-740 NC

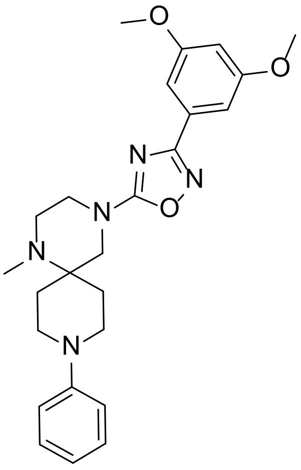

^{1}H NMR (600 MHz, DMSO-d_6) δ 7.21 (t, J = 8.6, 7.3 Hz, 2H), 7.01 (d, J = 2.3 Hz, 2H), 6.94 (d, J = 8.6 Hz, 2H), 6.76 (t, J = 7.3 Hz, 1H), 6.66 (t, J = 2.3 Hz, 1H), 3.78 (s, 6H), 3.66 – 3.62 (m, 2H), 3.62 (s, 2H), 3.48 (dt, J = 12.9, 4.5 Hz, 2H), 3.01 (ddd, J = 12.9, 10.4, 3.3 Hz, 2H), 2.82 – 2.75 (m, 2H), 2.31 (s, 3H), 1.94 (ddd, J = 14.0, 10.4, 4.5 Hz, 2H), 1.55 (dt, J = 14.0, 3.3 Hz, 2H).

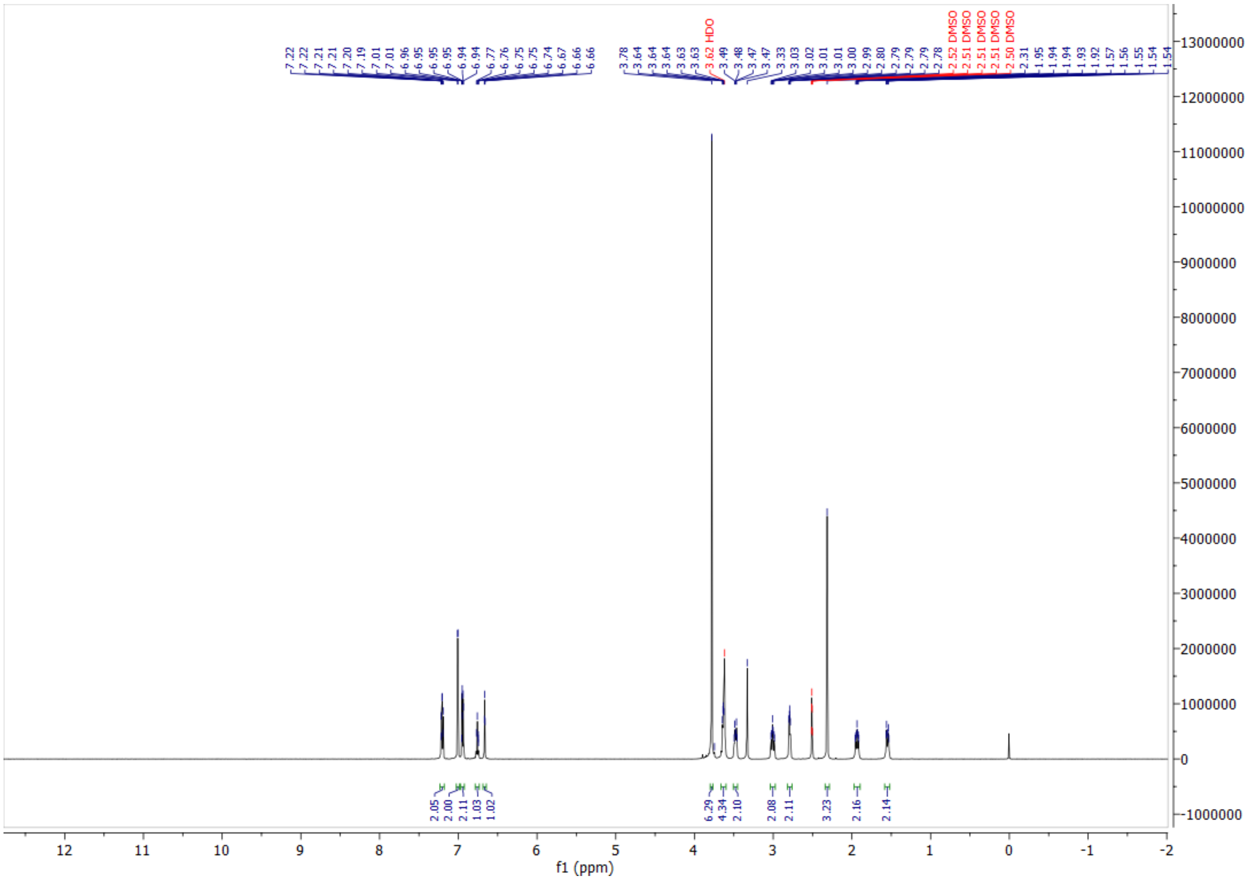

^{13}C NMR (151 MHz, DMSO) δ 171.25, 168.05, 161.17, 151.37, 129.57, 129.40, 118.89, 115.86, 105.00, 103.37, 55.86, 53.80, 49.62, 47.92, 44.98, 44.06, 40.55, 36.21, 27.81.

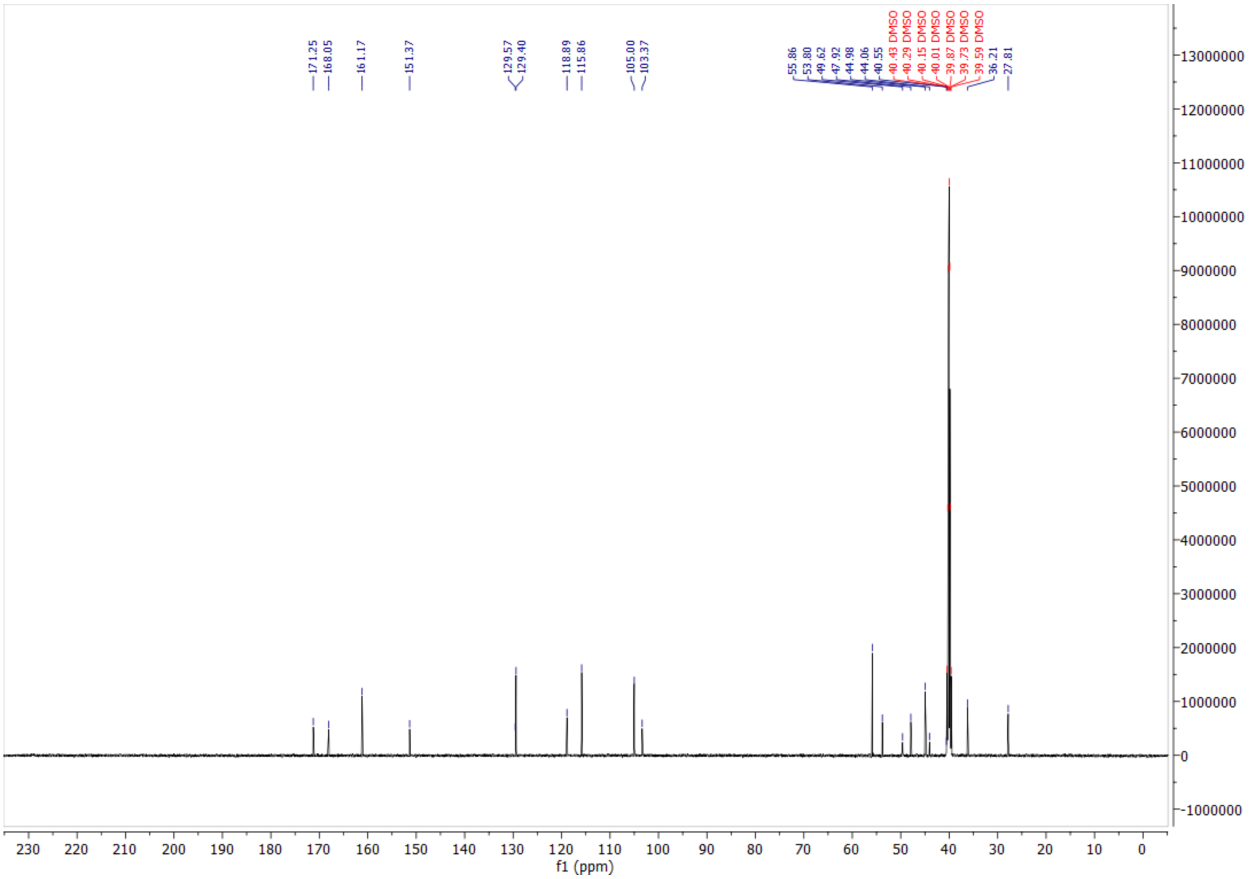

MRK-740

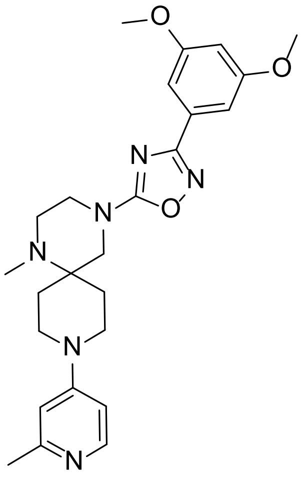

^{1}H NMR (600 MHz, DMSO-d_6) δ 8.02 (d, J = 6.0 Hz, 1H), 7.01 (dz, J = 2.3 Hz, 2H), 6.69 – 6.66 (m, 2H), 6.62 (dd, J = 6.0, 2.6 Hz, 1H), 3.79 (s, 6H), 3.65 – 3.62 (m, 2H), 3.62 – 3.57 (m, 4H), 3.19 (ddd, J = 13.1, 9.8, 3.3 Hz, 2H), 2.84 – 2.73 (m, 2H), 2.32 (s, 3H), 2.29 (s, 3H), 1.87 (ddd, J = 13.9, 9.8, 4.4 Hz, 2H), 1.52 (dt, J = 13.9, 4.4 Hz, 2H).

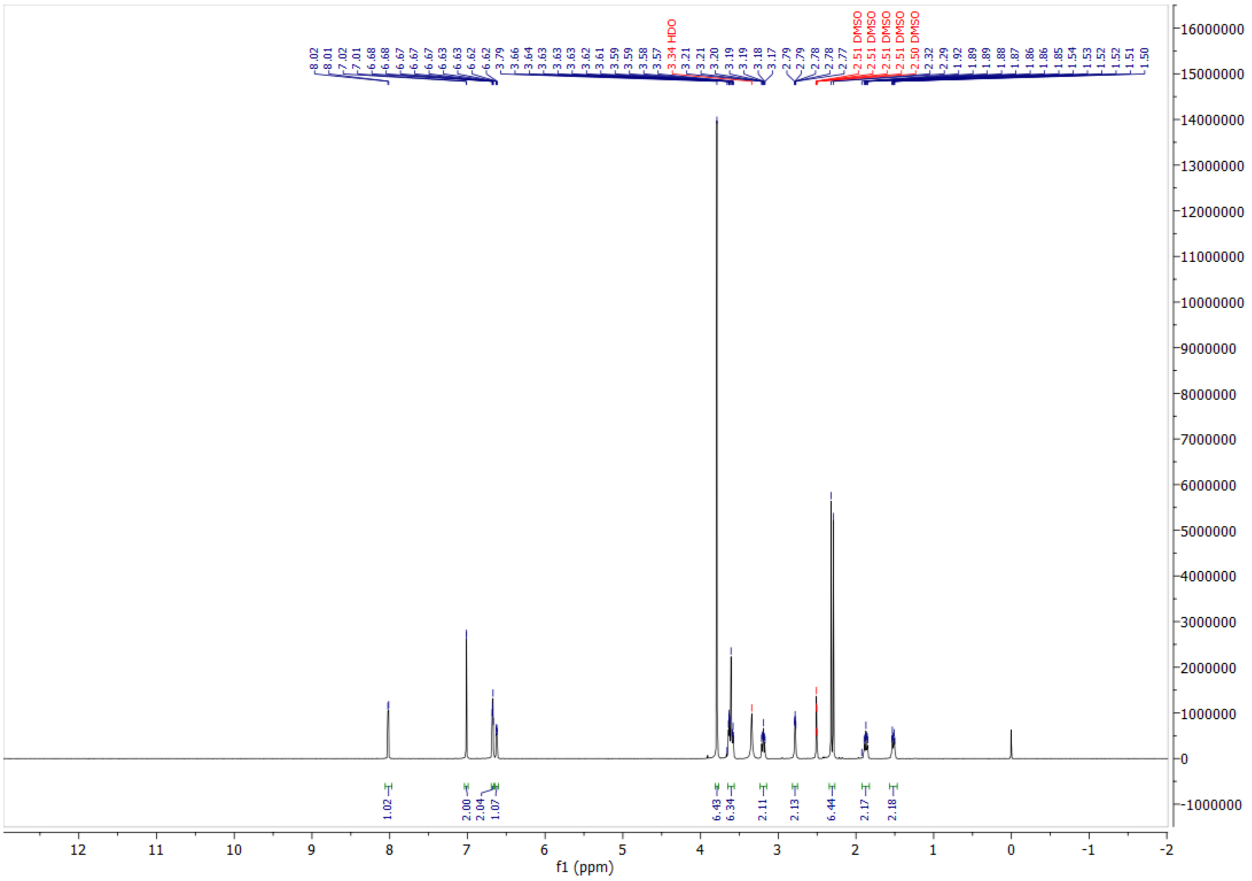

^{13}C NMR (151 MHz, DMSO) δ 171.27, 168.03, 161.17, 158.37, 155.02, 149.56, 129.53, 107.36, 106.25, 105.02, 103.33, 55.88, 53.90, 49.78, 47.88, 43.92, 42.24, 36.27, 27.44, 24.86.

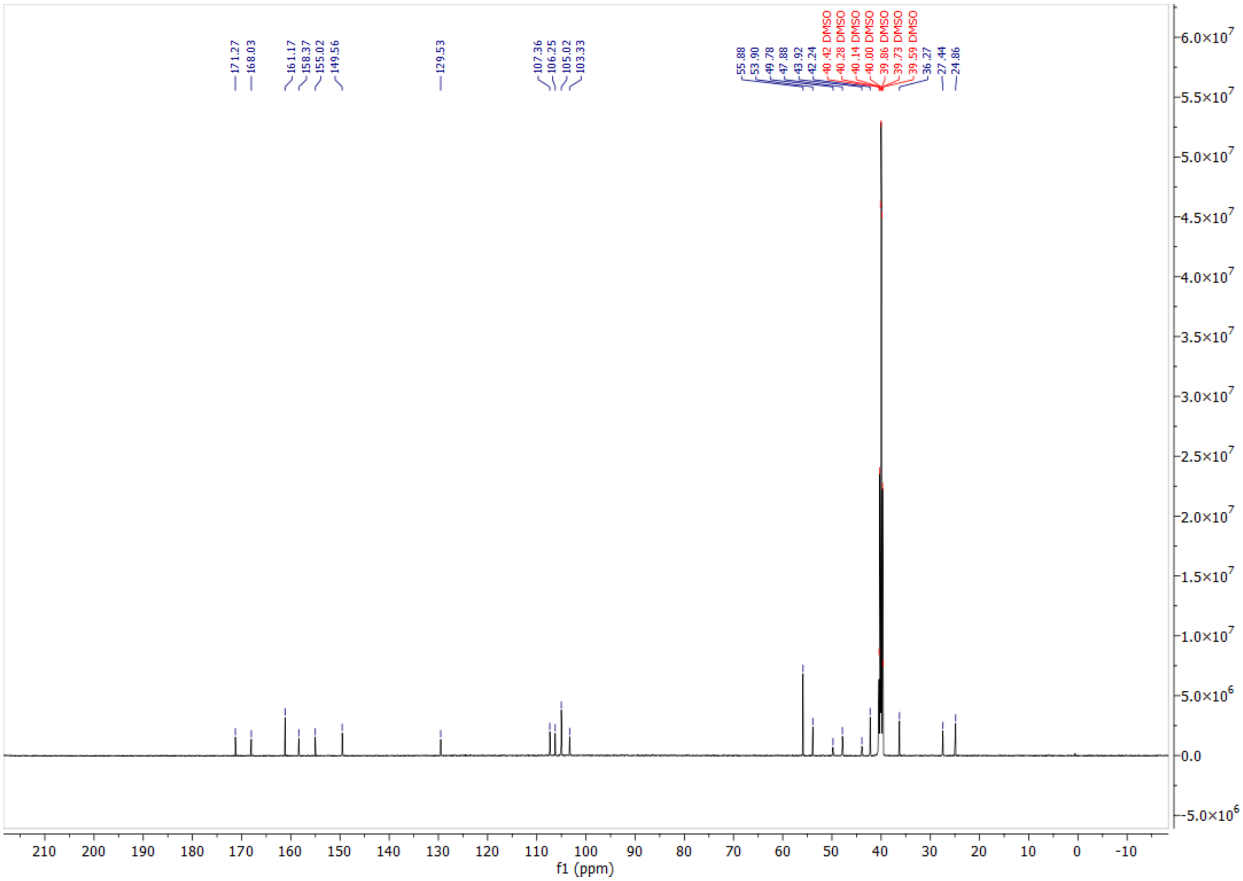

Supplement: Supplementary file 6 — Supplementary Data 3 [file 41467_2019_13652_MOESM6_ESM.pdf]
