# Supplementary material for: Discovery of a chemical probe for PRDM9
Source: Nat Commun. 2019 Dec 17;10:5759. doi: 10.1038/s41467-019-13652-x (PMC6917776; doi:10.1038/s41467-019-13652-x)
Supplement: Supplementary file 8 — Reporting Summary [file 41467_2019_13652_MOESM8_ESM.pdf]

nature research

Corresponding author(s): Benjamin Nicholson, Masoud Vedadi

Last updated by author(s): Nov 11, 2019

Reporting Summary

Nature Research wishes to improve the reproducibility of the work that we publish. This form provides structure for consistency and transparency in reporting. For further information on Nature Research policies, see Authors & Referees and the Editorial Policy Checklist.

Statistics

For all statistical analyses, confirm that the following items are present in the figure legend, table legend, main text, or Methods section.

n/a	Confirmed	
☐	☒	The exact sample size (*n*) for each experimental group/condition, given as a discrete number and unit of measurement
☐	☒	A statement on whether measurements were taken from distinct samples or whether the same sample was measured repeatedly
☐	☒	The statistical test(s) used AND whether they are one- or two-sided<br>*Only common tests should be described solely by name; describe more complex techniques in the Methods section.*
☒	☐	A description of all covariates tested
☒	☐	A description of any assumptions or corrections, such as tests of normality and adjustment for multiple comparisons
☐	☒	A full description of the statistical parameters including central tendency (e.g. means) or other basic estimates (e.g. regression coefficient) AND variation (e.g. standard deviation) or associated estimates of uncertainty (e.g. confidence intervals)
☐	☒	For null hypothesis testing, the test statistic (e.g. *F*, *t*, *r*) with confidence intervals, effect sizes, degrees of freedom and *P* value noted<br>*Give P values as exact values whenever suitable.*
☒	☐	For Bayesian analysis, information on the choice of priors and Markov chain Monte Carlo settings
☒	☐	For hierarchical and complex designs, identification of the appropriate level for tests and full reporting of outcomes
☒	☐	Estimates of effect sizes (e.g. Cohen's *d*, Pearson's *r*), indicating how they were calculated

Our web collection on statistics for biologists contains articles on many of the points above.

Software and code

Policy information about availability of computer code

Data collection	The data set was collected at the Advanced Photon Source beamline 24-ID-E with a wavelength of 0.979180 Å from a ADSC Quantum 315 CCD detector at 100 K. All data was processed and scaled with XDS33 and Aimless34. The structure was solved by molecular replacement using a trimmed structure of the human PRDM9 apo-enzyme (PDB accession code 4IJD, chain A, residues 201 - 354) as the search model with the program PhaserMR35. The stereochemical restraints for MRK-740 were generated using the program JLigand v1.0.4036. The structural models were refined using REFMAC537 and manually checked with COOT. Images were generated using PyMOL (The PyMOL Molecular Graphics System, v2.2.0, Schrödinger, LLC.).
Data analysis	The IC50 values were determined using GraphPad Prism 7 software. Kinetic curves for SPR analysis were fitted using a 1:1 binding model and the Biacore T200 Evaluation software (GE Health Sciences Inc.). Cell number was analysed with IncuCyte™ ZOOM (2015A) software. For cell-based thermal stability assays, Tm values were calculated using the drc_3.0-1 package and plots generated using tidyverse_1.2.1 and cowplot_0.9.4 packages in R version 3.5.1.

For manuscripts utilizing custom algorithms or software that are central to the research but not yet described in published literature, software must be made available to editors/reviewers. We strongly encourage code deposition in a community repository (e.g. GitHub). See the Nature Research guidelines for submitting code & software for further information.

Data

Policy information about availability of data

All manuscripts must include a data availability statement. This statement should provide the following information, where applicable:

- Accession codes, unique identifiers, or web links for publicly available datasets
- A list of figures that have associated raw data
- A description of any restrictions on data availability

The crystal structure dataset generated during and analyzed during the current study is available in the PDB repository (PDB ID: 6NM4).
All data generated and analyzed during this study are included in this article and its supplementary information files. Source data are provided for all relevant

Figures and Supplementary Figures.

Field-specific reporting

Please select the one below that is the best fit for your research. If you are not sure, read the appropriate sections before making your selection.

☒ Life sciences ☐ Behavioural & social sciences ☐ Ecological, evolutionary & environmental sciences

For a reference copy of the document with all sections, see nature.com/documents/nr-reporting-summary-flat.pdf

Life sciences study design

All studies must disclose on these points even when the disclosure is negative.

Sample size	No statistical method was used to pre-determine sample size. They sample sizes of each experiment were based on our and other's publications that is optimal to generate statistically significant results.
Data exclusions	No data were excluded
Replication	The data were generated from at least 3 technical replicates. All the details are indicated in the manuscript.
Randomization	No randomization was required
Blinding	No blinding was required

Reporting for specific materials, systems and methods

We require information from authors about some types of materials, experimental systems and methods used in many studies. Here, indicate whether each material, system or method listed is relevant to your study. If you are not sure if a list item applies to your research, read the appropriate section before selecting a response.

Materials & experimental systems

n/a	Involved in the study	
☐	☒	Antibodies
☐	☒	Eukaryotic cell lines
☒	☐	Palaeontology
☐	☒	Animals and other organisms
☒	☐	Human research participants
☒	☐	Clinical data

Methods

n/a	Involved in the study	
☒	☐	ChIP-seq
☒	☐	Flow cytometry
☒	☐	MRI-based neuroimaging

Antibodies

Antibodies used	Anti-GFP (#632381,Clontech), anti-H3K4me3(Millipore, #04-745), anti histone H3 (#ab10799, Abcam), anti-Flag (#F4799, Sigma)
Validation	All antibodies were validated by us using knockdown approach or by others in published peer reviewed papers.

Eukaryotic cell lines

Policy information about cell lines

Cell line source(s)	MCF7(ATCC® HTB-22™), HEK293T (kind gift from Sam Benchimol, York University), (MDA-MB436, ATCC® HTB130), 8226 and SKMM2 (kind gift from Rodger Tiedemann, Princess Margaret Cancer Centre), HCC1806 (ATCC®CRL 2335), HCC1143 (ATCC® CRL2321) and CAL851 (kind gift from Mathieu Lupien, Princess Margaret Cancer Centre)
Authentication	All cell lines were tested for authentication by STR profiling.
Mycoplasma contamination	All cell lines were mycoplasma negative, as determined by MycoAlert™ Mycoplasma Detection Kit(Lonza).
Commonly misidentified lines (See ICLAC register)	No commonly misidentified cell lines were used.

Animals and other organisms

Policy information about studies involving animals; ARRIVE guidelines recommended for reporting animal research

Laboratory animals	Prdm9-/- mice on a C57BL6 background, all males at P15 since we were studying meiotic prophase I in testis
Wild animals	No wild animal was used
Field-collected samples	No field-collected sample was used
Ethics oversight	Mice were housed under standard conditions, were maintained on a 12- hour light/dark cycle, were fed a standard chow diet containing 6% crude fat, and were treated in compliance with the institutional guidelines for animal care and use. All experimental protocols were approved by the Animal Care and Use Committee of Biological Resource Centre at Biopolis, A*STAR, Singapore (protocol#171268).

Note that full information on the approval of the study protocol must also be provided in the manuscript.
